# Unfilled gaps by polβ lead to aberrant ligation by LIG1 at the downstream steps of base excision repair pathway

**DOI:** 10.1093/nar/gkae104

**Published:** 2024-02-16

**Authors:** Mitchell Gulkis, Ernesto Martinez, Danah Almohdar, Melike Çağlayan

**Affiliations:** Department of Biochemistry and Molecular Biology, University of Florida, Gainesville, FL 32610, USA; Department of Biochemistry and Molecular Biology, University of Florida, Gainesville, FL 32610, USA; Department of Biochemistry and Molecular Biology, University of Florida, Gainesville, FL 32610, USA; Department of Biochemistry and Molecular Biology, University of Florida, Gainesville, FL 32610, USA

## Abstract

Base excision repair (BER) involves the tightly coordinated function of DNA polymerase β (polβ) and DNA ligase I (LIG1) at the downstream steps. Our previous studies emphasize that defective substrate-product channeling, from gap filling by polβ to nick sealing by LIG1, can lead to interruptions in repair pathway coordination. Yet, the molecular determinants that dictate accurate BER remains largely unknown. Here, we demonstrate that a lack of gap filling by polβ leads to faulty repair events and the formation of deleterious DNA intermediates. We dissect how ribonucleotide challenge and cancer-associated mutations could adversely impact the ability of polβ to efficiently fill the one nucleotide gap repair intermediate which subsequently results in gap ligation by LIG1, leading to the formation of single-nucleotide deletion products. Moreover, we demonstrate that LIG1 is not capable of discriminating against nick DNA containing a 3′-ribonucleotide, regardless of base-pairing potential or damage. Finally, AP-Endonuclease 1 (APE1) shows distinct substrate specificity for the exonuclease removal of 3′-mismatched bases and ribonucleotides from nick repair intermediate. Overall, our results reveal that unfilled gaps result in impaired coordination between polβ and LIG1, defining a possible type of mutagenic event at the downstream steps where APE1 could provide a proofreading role to maintain BER efficiency.

## Graphical Abstract

**Graphical Abstract:**
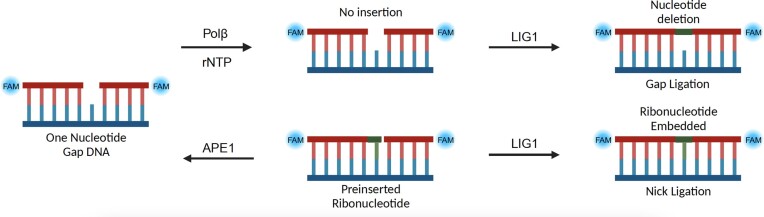

## Introduction

Base excision repair (BER) is a critical process for repairing base lesions and abasic sites, thereby preventing the mutagenic and lethal consequences of DNA damage (1–3). The BER pathway involves a series of sequential enzymatic steps which requires direct transfer of repair intermediates from one enzyme to the next in the pathway, in a process referred to as substrate-product channeling, to prevent the accumulation of toxic strand break intermediates in cells (3–5). In short-patch (SP)-BER, a damaged base is first removed by a lesion-specific DNA glycosylase, resulting in an apurinic/apyrimidinic (AP)-site in double-stranded DNA, which is then recognized and cleaved by AP-Endonuclease 1 (APE1), generating a single-strand break with 3′-OH and 5′-deoxyribose phosphate (5′-dRP) ends (1,2) Next, DNA polymerase (pol) β removes the 5′-dRP group via its lyase activity and subsequently catalyzes template-directed gap filling DNA synthesis through its nucleotidyl transferase activity (1,2). BER is completed with a final DNA ligation step by DNA ligase (LIG) 1 or LIG3α that catalyzes phosphodiester bond formation between 3′-OH and 5′-P ends of the nick (1,2). Studies indicate that LIG1 and LIG3α can be interchangeable in BER, apparently being selected, at least in part, by the choice between either the SP- or long-patch (LP)-BER subpathway (3). After initial damage recognition and its processing by DNA glycosylase and APE1 activities, respectively, the nick repair product generated by polβ following correct nucleotide incorporation into gap DNA is channeled to LIG1 or LIG3α for its subsequent nick sealing during the downstream steps of the BER pathway (3). Our biochemical and structural studies have revealed that, in certain situations, BER responses can lead to mutagenic repair where the gap filling coupled to the DNA ligation steps of the repair pathway can be compromised (6–9).

Polβ is an error-prone polymerase that incorporates mismatches approximately one out of every 5000 nucleotide insertion events during BER (10). Ribonucleotide triphosphates (rNTPs) are much more abundant than deoxyribonucleotide triphosphates (dNTPs) in human cells, and rNTP misincorporation by DNA polymerases constitutes a major source of ribonucleotides embedded into genomic DNA, which could make the phosphodiester backbone more labile to DNA damage, influence DNA conformation, increase the mutation rate and hinder replication fork progress (11–15). However, the impact of ribonucleotides on BER pathway coordination at the downstream steps involving rNTP incorporation by error-prone polβ and subsequent nick sealing by DNA ligase remains undefined.

Moreover, 30% of a variety of human tumors such as lung, gastric, colorectal and prostate cancer have been found to express polβ variants carrying single amino acid substitutions in distinct regions of the protein (16–18). These polβ cancer-associated variants possess aberrant activity *in vitro* such as diminished fidelity, stemming from reduced discrimination against incorrect nucleotides, leading to mutator phenotypes (Y265C and E288K), slower rate of dRP lyase (L22P) or polymerase (S229L) activity and a complete lack of gap filling activity (E295K) (17–30). Previous studies have also shown that the expression of these polβ variants in mouse or human cells leads to cancer phenotypes such as an increased mutation frequency, permanent cellular transformation, aberrant DNA repair, accumulation of toxic BER intermediates and ultimately genomic instability, suggesting a pivotal role for polβ-mediated high-fidelity DNA synthesis in carcinogenesis. (17,20,22,24,27,28). Yet, how the efficiency of substrate–product channeling at the downstream steps of BER could be adversely affected in case of unfilled gap mediated by polβ cancer-associated variants remains entirely unknown.

In the present study, we comprehensively investigated the impact of canonical, mismatched, damaged and ribonucleotide incorporation by polβ on BER pathway coordination between gap filling by polβ and DNA ligation by LIG1. Our findings revealed that while polβ is inefficient at inserting dNTP and rNTP mismatches, LIG1 seals the one nucleotide gap repair intermediate itself, referred to as gap ligation, resulting in the formation of deletion mutagenesis products. Upon mutation of the steric gate residue of polβ (Y271A), we observed efficient 8oxorGTP incorporation and its subsequent mutagenic ligation, while LIG1 fails following polβ 8oxodGTP insertion, resulting in the formation of abortive ligation product. Moreover, our findings demonstrated that polβ cancer-associated variants Y265C, E288K and E295K leave the gap repair intermediate unfilled even in the presence of correct nucleotides, which leads to gap ligation by LIG1 and the formation of deletion mutagenesis products.

In the current work, we also extensively characterized the substrate specificity of LIG1 and APE1 to elucidate the efficiency of nick sealing versus proofreading at the final steps of the BER pathway for a variety of nick repair intermediates containing canonical, mismatched, damaged or ribonucleotides at the 3′-end. Our results demonstrated that LIG1 cannot discriminate against a ‘wrong’ sugar and is able to ligate almost all possible 12 ribonucleotide mismatches, while it shows distinct efficiency for the ligation of deoxyribonucleotide mismatches. Finally, we showed that APE1 can remove a 3′-base through its proofreading exonuclease activity from the nick DNA substrates containing ribonucleotides or mismatches depending on the base-pairing architecture. Furthermore, LIG1 can seal the nick repair intermediate with 3′-8oxodG or 3′-8oxorG opposite A from which APE1 is able to remove the damaged base efficiently as well.

Overall, our findings revealed that ribonucleotide discrimination by polβ is the key determinate which prevents ribonucleotides from interfering with BER pathway coordination resulting in faulty repair events or deleterious DNA intermediates, and APE1/LIG1 interplay plays a critical role for ensuring accurate repair at the downstream steps. Additionally, the present study demonstrates that unfilled gaps left by polβ, wild-type or cancer-associated mutants, are erroneously ligated by LIG1, resulting in a deviation from canonical BER pathway coordination and the formation of aberrant repair intermediates with one nucleotide deletions.

## Materials and methods

### Preparation of DNA substrates

Oligodeoxyribonucleotides with and without a 6-carboxyfluorescein (FAM) label were obtained from Integrated DNA Technologies. One nucleotide gap DNA substrates with template base A or C and a FAM label at the 5′-end were used in polβ nucleotide insertion assays (Supplementary Table S1). One nucleotide gap DNA substrates with template base A or C and FAM labels at both 3′- and 5′-ends were used in polβ nucleotide insertion coupled to DNA ligation assays (Supplementary Table S2). Nick DNA substrates with preinserted 3′-deoxyribo- or ribo-nucleotide mismatches and FAM label at the 3′-end were used in DNA ligation assays (Supplementary Tables S3 and S4). Nick DNA substrates with preinserted 3′-deoxyribo- or ribo-nucleotide mismatches and FAM label at the 5′-end were used in APE1 exonuclease assays (Supplementary Tables S5 and S6).

### Protein purifications

Human wild-type his-tag LIG1 full-length (1-919) was overexpressed in Rosetta (DE3) pLysS *Escherichia coli* (*E. coli*) cells and grown in Terrific Broth (TB) media with kanamycin (50 μg ml^−1^) and chloramphenicol (34 μg ml^−1^) at 37°C. Once the OD_600_ reached 1.0, the cells were induced with 0.5 mM isopropyl β-D-thiogalactoside (IPTG), and overexpression continued overnight at 20°C. After centrifugation, the cells were lysed in lysis buffer containing 50 mM Tris-HCl (pH 7.0), 500 mM NaCl, 20 mM imidazole, 2 mM β-mercaptoethanol, 5% glycerol and 1 mM Phenylmethylsulfonyl fluoride (PMSF) by sonication at 4°C. The cell lysate was pelleted at 31 000 × *g* for 90 min at 4°C. The cell lysis solution was filter clarified and then loaded onto a HisTrap HP column that was previously equilibrated with binding buffer containing 50 mM Tris-HCl (pH 7.0), 500 mM NaCl, 20 mM imidazole, 2 mM β-mercaptoethanol and 5% glycerol. The column was washed with binding buffer and then eluted with an imidazole gradient (0–500 mM) at 4°C. The collected fractions were then subsequently loaded onto a HiTrap Heparin column that was equilibrated with binding buffer containing 20 mM Tris-HCl (pH 7.0), 50 mM NaCl, 2 mM β-mercaptoethanol and 5% glycerol, and the protein was eluted with a linear gradient of NaCl up to 1 M. LIG1 protein was further purified by a Superdex 200 gel filtration column in buffer containing 50 mM Tris-HCl (pH 7.0), 200 mM NaCl, 1 mM DTT and 5% glycerol. Human DNA ligase (LIG) 3α (pET-24b) full-length (1-922) protein was overexpressed in BL21(DE3) *E. coli* cells in LB media at 37°C for 8 h and induced with 0.5 mM IPTG. The cells were harvested, lysed at 4°C, and then clarified as described above. The supernatant was loaded onto HisTrap HP column and purified with an increasing imidazole gradient (0–300 mM) elution at 4°C. The collected fractions were then further purified by Superdex 200 Increase 10/300 chromatography in the buffer containing 50 mM Tris-HCl, pH 7.0, 500 mM NaCl, glycerol 5%, 1 mM DTT.

Human wild-type full-length (1–335) polβ with a GST-tag (pGEX-6p-1) was overexpressed in BL21(DE3) pLysS *E. coli* cells in TB media at 37°C. When the OD_600_ reached 1.0, the cells were induced with 0.5 mM IPTG, and the overexpression continued overnight at 20°C. After cell lysis at 4°C by sonication in lysis buffer containing 1× PBS (pH 7.3), 200 mM NaCl, 1 mM Dithiothreitol (DTT) and 1 mM PMSF, the cell lysate was pelleted at 31 000 × *g* for 90 min and then filter clarified. The clarified supernatant was loaded onto a GSTrap HP column, washed with lysis buffer, and eluted with elution buffer containing 50 mM Tris-HCl (pH 8.0), and 10 mM reduced glutathione. To cleave the GST-tag, the recombinant polβ protein was incubated with PreScission Protease for 16 h at 4°C in buffer containing 1× PBS (pH 7.3), 200 mM NaCl and 1 mM DTT. Polβ protein was then subsequently passed through a preequilibrated GSTrap HP column to remove the cleaved tag, and the protein without the GST-tag was then further purified by loading onto a Heparin HP column that was equilibrated with binding buffer containing 20 mM Tris-HCl (pH 7.0), 50 mM NaCl, 2 mM β-mercaptoethanol and 5% glycerol. The protein was then eluted using a linear gradient up to 1 M NaCl. Finally, polβ was purified by a Superdex 200 gel filtration column in buffer containing 50 mM Tris-HCl (pH 7.0), 200 mM NaCl, 1 mM DTT and 5% glycerol. Plasmid DNA coding Y271A (pGEX-6p-1), as well as Y265C, E288K and E295K mutations (pGEX-4T-3) were generated by site directed mutagenesis and the coding sequences of the mutants were confirmed by DNA sequencing prior to purification. The steric gate mutant protein, polβ Y271A, was overexpressed and purified as described above. Polβ cancer-associated mutants Y265C, E288K and E295K were purified as described above except the GST-tag cleavage step.

Human his-tag wild-type full-length (1-335) APE1 was overexpressed in BL21(DE3) *E. coli* cells in Lysogeny broth (LB) media at 37°C. When the OD_600_ reached 1.0, the cells were induced with 0.5 mM IPTG, and the overexpression continued overnight at 28°C. After centrifugation, the cells were lysed in lysis buffer containing 50 mM Tris-HCl (pH 7.0), 500 mM NaCl, 20 mM imidazole, 2 mM β-mercaptoethanol, 5% glycerol and 1 mM PMSF by sonication at 4°C. The lysate was pelleted at 31 000 × *g* for 90 min at 4°C and then the supernatant was filter clarified. The clarified supernatant was loaded onto a HisTrap HP column and eluted with an increasing imidazole gradient (0–300 mM) at 4°C. The collected fractions were then subsequently loaded onto a HiTrap Heparin column and eluted with a linear gradient of NaCl up to 1 M. The recombinant APE1 protein was then further purified by a Superdex 200 gel filtration column in buffer containing 20 mM Tris-HCl (pH 7.0), 200 mM NaCl and 1 mM DTT.

All proteins purified in this study were stored in aliquots at –80°C. Protein quality was evaluated on a 10% SDS-PAGE gel, and protein concentrations were measured using the absorbance at 280 nm.

### Nucleotide insertion assays

Polβ nucleotide incorporation assays were performed to examine nucleotide insertion efficiency of polβ into gap DNA containing template base A or C. The reaction mixture contains 50 mM Tris-HCl (pH 7.5), 100 mM KCl, 10 mM MgCl_2_, 1 mM ATP, 1 mM DTT, 100 μg ml^−1^ BSA, 10% glycerol, DNA substrate (500 nM), and dNTP, rNTP, 8-oxodGTP or 8-oxorGTP (10 μM) in a final volume of 10 μl. The reaction was initiated by the addition of polβ (wild-type, Y271A, Y265C, E288K or E295K) at a final concentration of 100 nM, and incubated at 37°C for the times as indicated in the figure legends. The reaction products were then quenched by mixing with an equal amount of gel loading buffer (95% formamide, 20 mM EDTA, 0.02% bromophenol blue and 0.02% xylene cyanol) and then separated by electrophoresis on an 18% polyacrylamide gel. The gels were scanned with a Typhoon PhosphorImager (Amersham Typhoon RGB), and the data were analyzed using ImageQuant software as described previously.

### Nucleotide insertion coupled to DNA ligation assays

Coupled assays were used to measure polβ nucleotide insertion and DNA ligation in the same reaction simultaneously using one nucleotide gap DNA substrates containing template base A or C. The reaction mixture contains 50 mM Tris-HCl (pH 7.5), 100 mM KCl, 10 mM MgCl_2_, 1 mM ATP, 1 mM DTT, 100 μg ml^−1^ BSA, 10% glycerol, DNA substrate (500 nM) and dNTP, rNTP, 8-oxodGTP or 8-oxorGTP (10 μM) in a final volume of 10 μl. The reaction was initiated by the addition of pre-incubated enzyme mixture of LIG1 and polβ (wild-type, Y271A, Y265C, E288K or E295K) at a final concentration of 100 nM each, and incubated at 37°C for the times as indicated in the figure legends. The reaction samples were then quenched by mixing with an equal volume of the loading dye. The products were separated, and the data were analyzed as described above. The coupled assays were performed similarly in the presence of polβ, rNTP and Lig3α.

### DNA ligation assays

DNA ligation assays were performed to evaluate the substrate specificity of LIG1 using nick DNA substrates containing 3′-preinserted deoxyribo- or ribonucleotide mismatches and 3′-8oxodG or 3′-8oxorG. The ligation reaction mixture contains 50 mM Tris-HCl (pH 7.5), 100 mM KCl, 10 mM MgCl_2_, 1 mM ATP, 1 mM DTT, 100 μgml^−1^ BSA, 10% glycerol and DNA substrate (500 nM) in a final volume of 10 μl. The reaction was initiated by the addition of LIG1 (100 nM), incubated at 37°C and quenched at the time points as indicated in the figure legends. The products were separated, and the data were analyzed as described above.

### APE1 exonuclease assays

Exonuclease assays were performed to evaluate the substrate specificity of APE1 using nick DNA substrates containing 3′-preinserted deoxyribo- or ribonucleotide mismatches and 3′-8oxodG or 3′-8oxorG. The reaction mixture contains 50 mM Tris-HCl (pH 7.5), 100 mM KCl, 10 mM MgCl_2_, 1 mM ATP, 1 mM DTT, 100 μg ml^−1^ BSA, 10% glycerol and DNA substrate (500 nM) in a final volume of 10 μl. The reaction was initiated by the addition of APE1 (1 μM) and incubated at 37°C for the time points as indicated in the figure legends. The products were separated, and the data were analyzed as described above.

### DNA-binding measurements by BioLayer Interferometry assay

DNA-binding kinetics of polβ, LIG1 and LIG3α was measured by BioLayer Interferometry (BLI) assays in real time using the Octet QKe (Fortebio) using one nucleotide gap DNA substrate with 3′-biotin label. Streptavidin (SA) biosensors were used to attach the biotin labeled DNA. BLI experiments were performed at 20°C in 96-well microplates with agitation set to 1000 rpm. The SA biosensors were hydrated at 20ºC for 20 min in the buffer containing 50 mM Tris-HCl pH 7.5, 100 mM KCl, and 1 mM DTT. The sensors were then immersed in DNA (40 nM) in the buffer for 300 s. After recording an initial baseline for 60 s, the sensors with DNA were exposed to the concentration range of the proteins as indicated in the figure legends. DNA binding was performed for 240 s association, and then for 240 s dissociation. In all measurements, the affinity constants (*K*_D_), the association (*k*_on_) and dissociation (*k*_off_) rates were calculated using the ForteBio Data Analysis software with 1:1 binding model. The association rate = *k*_on_ [ligand][analyte] and the dissociation rate = *k*_off_ [ligand-analyte]. At equilibrium, forward and reverse rates are equal. All images were drawn using GraphPad Prism 7.

## Results

### Impact of polβ ribomismatch incorporation on DNA ligation

**Figure 1.**
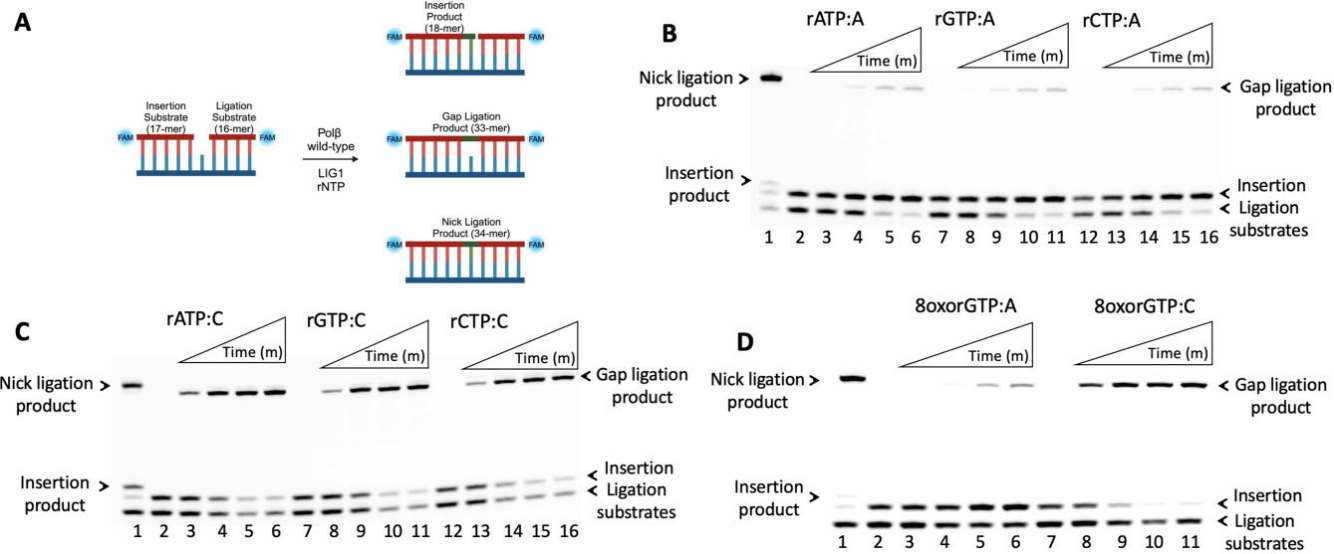
Ligation efficiency of polβ ribonucleotide insertion by LIG1. (**A**) Scheme shows the substrate and products observed in the coupled assay. (**B**and**C**) Lane 1 is the positive control showing the ligation of polβ wild-type dTTP:A (B) and dGTP:C (C) insertion products by LIG1. Lanes 2, 7 and 12 are the negative enzyme controls of gap DNA substrates containing template base A or C. Lanes 3–6, 8–11 and 13–16 are the reaction products in the presence of polβ wild-type, LIG1 and rNTP as indicated in the figure, and correspond to time points of 0.5, 1, 3 and 5 min. Representative gel images of three independent repeats. (**D**) Lane 1 is the positive control showing the ligation of polβ wild-type dTTP:A insertion product by LIG1. Lanes 2 and 7 are the negative enzyme controls of gap DNA substrates containing template base A or C. Lanes 3–6 and 8–11 are the reaction products in the presence of polβ wild-type, LIG1 and 8-oxorGTP as indicated in the figure, and correspond to time points of 0.5, 1, 3 and 5 min. Representative gel images of three independent repeats.

We first investigated the impact of polβ ribonucleotide insertion on BER pathway coordination at the downstream steps by performing coupled assays containing polβ wild-type, LIG1, rNTP and one nucleotide gap DNA substrate (Figure 1A). For ribonucleotide mismatches rATP, rGTP and rCTP, we mainly observed ligation products of the one nucleotide gap DNA substrate, referred to as gap ligation hereafter (Figure 1B,C). These gap ligation products were more abundant in reactions including polβ and gap DNA substrate with template base C (Figure 1C) over template base A (Figure 1B). Gap versus nick ligation was revealed by the difference in the size of the products between the ligation of gap DNA itself versus nick sealing after polβ correct nucleotide insertions dTTP:A and dGTP:C by LIG1 in the control reactions (Figure 1B and C, lane 1). Consistent with previous reports (31–33), we also showed inefficient incorporation of rNTPs by polβ (Supplementary Figure S1).

Ribonucleotides are susceptible to chemical modification, through a variety of mechanisms, which causes damage to the structure and function of the ribonucleotide (34,35). A common form of ribonucleotide damage is oxidative damage, caused by endogenous or exogenous reactive oxygen species, which leads to oxidized ribonucleotides such as 8-oxo-guanosine-5′-triphosphate (8oxorGTP) (36,37). As demonstrated in the X-ray crystal structure of polβ, the active site can accommodate 8oxorGTP opposite templating C and mutagenic A in *anti*- and *syn*-conformation, respectively (31). In the present study, we also investigated the ligation efficiency following polβ oxidized ribonucleotide incorporation in coupled reactions including polβ wild-type, 8oxorGTP and LIG1. Our results demonstrated that LIG1 attempts to ligate the one nucleotide gap DNA substrate, creating gap ligation products (Figure 1D). We additionally observed more abundant gap ligation products in the presence of the gap DNA substrate with template C over template A, similar to ribonucleotide mismatch reactions (Figure 1D, lanes 3–6 versus 8–11). In the insertion assays containing polβ alone, our results are consistent with our coupled assays which show that polβ wild-type is inefficient at inserting 8oxorGTP (Supplementary Figure S1). We performed the same experiments with LIG3α and obtained similar results showing the gap ligation products in the presence of polβ and rNTP and the preference for template base C over A (Supplementary Figure S2).

### Impact of polβ steric-gate mutant on the mutagenic nick sealing of ribonucleotide incorporation products

The discrimination against ribonucleotide by repair and replication DNA polymerases is essential for the maintenance of genome integrity (11–14). Previous structure/function studies have reported the steric gate role of the Tyr(Y)271 backbone carbonyl of polβ, which clashes with the ribose 2′-OH of the incoming rNTP and discourages polβ from incorporating ribonucleotides (31–33). Furthermore, it has been shown that the Y271A mutation leads to ∼12-fold increase in ribonucleotide insertion by polβ as demonstrated by a >10-fold loss in sugar discrimination and an increase in the insertion of 8oxorGTP:A, having a similar efficiency to a non-damaged matched ribonucleotide (31–33)

**Figure 2.**
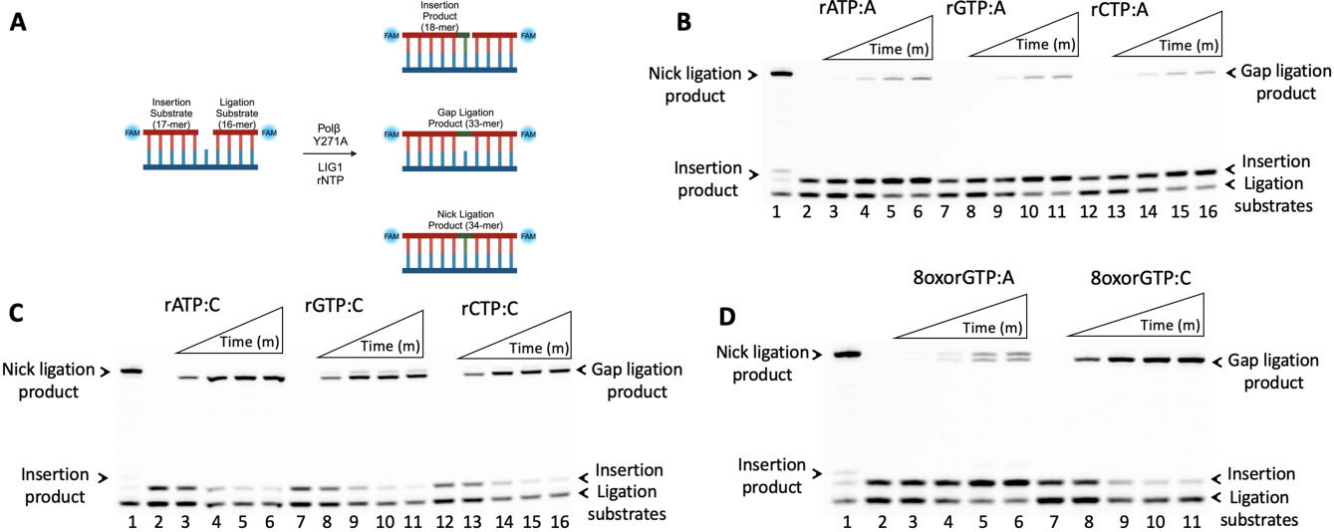
Ligation efficiency of polβ steric gate mutant Y271A ribonucleotide insertions by LIG1. (**A**) Scheme showing the substrate and products observed in the coupled assay. (**B-C**) Lane 1 is the positive control showing the ligation of polβ Y271A dTTP:A (B) and dGTP:C (C) insertion products by LIG1. Lanes 2, 7 and 12 are the negative enzyme controls of gap DNA substrates containing template base A or C. Lanes 3–6, 8–11 and 13–16 are the reaction products in the presence of polβ Y271A, LIG1 and rNTP as indicated in the figure, and correspond to time points of 0.5, 1, 3 and 5 min. Representative gel images of three independent repeats. (**D**) Lane 1 is the positive control showing the ligation of polβ Y271A dTTP:A insertion product by LIG1. Lanes 2 and 7 are the negative enzyme controls of gap DNA substrates containing template base A or C. Lanes 3–6 and 8–11 are the reaction products in the presence of polβ Y271A, LIG1, and 8-oxorGTP as indicated in the figure, and correspond to time points of 0.5, 1, 3 and 5 min. Representative gel images of three independent repeats.

In order to compare the impact of increasing the ribonucleotide tolerance of polβ on the nick sealing efficiency at the final steps of the BER pathway, we next investigated the ligation of rNTP and 8oxorGTP incorporations by polβ Y271A in coupled assays as described above (Figure 2A). Our results showed the formation of gap ligation products (Figure 2B-C), which was similar to the ligation of gap DNA itself we observed with polβ wild-type (Figure 1). We obtained slightly more nick ligation products in the coupled reaction including canonical rNTP:templating base pair rGTP:C (Figure 2C, lanes 8–11). Similarly, we observed both gap and nick ligation products for 8oxorGTP:A (Figure 2D, lanes 2–6). Even with increased ribonucleotide tolerance, there was no nick ligation products for 8oxorGTP:C (Figure 2D, lanes 8–11). The insertion results with polβ Y271A alone demonstrated that upon increased ribonucleotide tolerance, polβ is capable of inserting rGTP opposite C as well as 8oxorGTP opposite template A more efficiently (Supplementary Figure S3). Furthermore, we showed similar nick sealing efficiency by LIG1 after correct dGTP:C insertions by polβ wild-type versus the Y271A mutant in control coupled assays, demonstrating that the Y271A mutation does not effect the canonical gap filling activity of polβ and LIG1 can seal resulting nick repair product efficiently (Supplementary Figure S4).

Overall, our findings demonstrate the formation of single deletion mutagenesis intermediates that could be formed due to unfilled gaps by polβ in the presence of rNTPs or 8oxorGTP when LIG1 attempts ligating the gap repair intermediate at the downstream steps of the BER pathway. These findings also highlight the importance of ribonucleotide discrimination by polβ for accurate BER pathway coordination.

### Comparison of ligation efficiency after polβ mismatch incorporation

To compare the impact of deoxyribo- versus ribo-nucleotide mismatch insertions and subsequent DNA ligation at the downstream steps of the BER pathway, we also analyzed the nick sealing efficiency by LIG1 after polβ dNTP mismatch insertions in coupled assays (Supplementary Figure S5A).

As we previously reported (7), our results demonstrated that LIG1 cannot ligate the nick repair products of polβ mismatch insertions opposite template base A or C (Supplementary Figure S5B and C). Similar to the coupled assays including rNTP mismatches, in both cases, the products of polβ dNTP mismatch insertion coupled to ligation were mainly the ligation products of the one nucleotide gap DNA itself, as revealed by the difference with the nick ligation products after polβ correct dTTP:A or dGTP:C insertion. We previously demonstrated that oxidized nucleotide incorporation by polβ confounds DNA ligase resulting in the accumulation of cytotoxic ligation failure products (6). Consistent with this report, our results showed a failure in the ligation along with mutagenic nick sealing after polβ 8-oxodGTP insertion opposite template base A (Supplementary Figure S5D). Furthermore, polβ insertion assays showed an inefficiency of inserting dNTP mismatches opposite A or C, consistent with what we observed in our coupled assays (Supplementary Figure S6).

### Inefficient insertion of correct nucleotides by polβ cancer-associated mutants leads to gap ligation of unfilled gaps by LIG1

**Figure 3.**
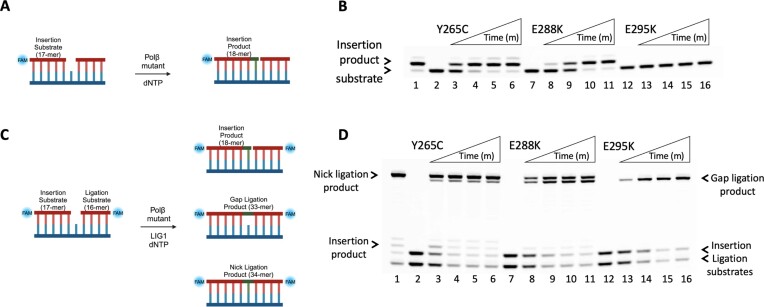
Impact of polβ cancer-associated mutants on the ligation of correct nucleotide insertion products by LIG1. (**A**) Scheme shows the substrate and product observed in the insertion assay. (**B**) Lane 1 is the positive control showing dGTP:C insertion product by polβ. Lanes 2, 7 and 12 are the negative enzyme controls of gap DNA substrates containing template base C. Lanes 3–6, 8–11 and 13–16 are the insertion products by polβ mutants Y265C, E288K, E295K, respectively, and correspond to time points of 0.5, 1, 3 and 5 min. Representative gel images of three independent repeats. (**C**) Scheme shows the substrate and products observed in the coupled assay. (**D**) Lane 1 is the positive control showing the ligation of polβ dGTP:C insertion products by LIG1. Lanes 2, 7 and 12 are the negative enzyme controls of gap DNA substrates containing template base C. Lanes 3–6, 8–11 and 13–16 are the reaction products in the presence of polβ mutants Y265C, E288K, E295K, respectively, and correspond to time points of 0.5, 1, 3 and 5 min. Representative gel images of three independent repeats.

It has been extensively reported that germline and tumor-associated variants of polβ catalyze aberrant BER that leads to genomic instability (16–30). One of these polβ variants, E295K, which has been identified in both gastric and colon carcinoma, appears to lack DNA polymerase activity while showing the same level of dRP lyase function and gap DNA binding affinity as the wild-type enzyme (19,21,22). The polβ E295K variant interferes with BER and cannot fill single-nucleotide gaps in an efficient manner, leading to the accumulation of BER intermediates, formation of double-strand breaks, induced cellular transformation and an increase in sister chromatid exchanges (19,21,22). Furthermore, polβ cancer-associated variants that exhibit mutator phenotypes, Y265C and E288K, have been previously shown to affect the enzyme’s ability to discriminate between correct and incorrect dNTPs, resulting in filling gaps in an error-prone manner with increased mutagenesis *in vitro* and also inducing cellular transformation when expressed in mouse cells, leading to genome instability (18,23–25). In light of our observations above demonstrating gap ligation in the case of inefficient incorporation of dNTP or rNTP mismatches by polβ wild-type, in the present study, we examined the impact of correct nucleotide insertions by polβ cancer-associated variants Y265C, E288K and E295K on nick sealing by LIG1 in coupled assays. Under our reaction conditions, polβ dGTP:C nucleotide insertion assays showed moderate gap filling for Y265C and E288K, and no detectable gap filling activity for E295K (Figure 3A,B).

In the coupled assay including polβ and LIG1 (Figure 3C), we demonstrated that the E295K mutant is unable to insert a correct nucleotide, and that LIG1 seals the one nucleotide gap DNA itself (Figure 3D, lanes 13–16). Interestingly, we observed both gap ligation and nick ligation products for polβ Y265C and E288K variants (Figure 3D, lanes 3–6 and 8–11, respectively). More gap ligation product was formed in the coupled reaction including E288K, which could be due to less efficient dGTP:C insertion by this polβ variant (Figure 3B, lines 8–11). This is consistent with our coupled results containing polβ wild-type with rNTP or dNTP mismatch, which show no insertion and gap ligation products only (Figures 1-2). Taken together, these results could mimic cellular conditions in which polβ leaves gaps unfilled during the DNA synthesis step of the BER pathway, such as in gastric carcinoma cancer patients carrying the somatic E295K mutation in the *POLB* gene, where canonical repair pathway coordination could be confounded at the downstream steps. Additionally, in cellular contexts where the gap filling activity of polβ is slower, such as in the colorectal cancer patients with E288K mutation or as shown in Lupus disease with polβ Y265C somatic mutation, LIG1 can attempt to ligate the gap leading to the formation of aberrant repair intermediate.

### Gap ligation of one nucleotide gap DNA by LIG1

To understand whether gap ligation of one nucleotide gap DNA (Figures 1–3) is dependent on polβ or free nucleotide (rNTP or dNTP), we performed ligation assays containing only LIG1 and the one nucleotide gap DNA substrate (Supplementary Figure S7A). Our results demonstrated that LIG1 exhibits different efficiency to seal the gap depending on the identity of the template base (Supplementary Figure S7B). Interestingly, we observed much more efficient gap ligation when the template base was C compared to A, T or G (Supplementary Figure S7C). This suggests that gap ligation by LIG1 demonstrates substrate specificity. We observed similar efficiency of gap ligation in the ligation versus coupled reactions with the polβ E295K variant that is deficient in gap filling activity (Figure 3) as well as coupled reactions with polβ wild-type in the presence of either rNTPs or dNTP mismatches (Figure 1). Furthermore, we demonstrated that LIG3α can also seal the gap itself as efficient as LIG1 (Supplementary Figure S7D). Taken together, this suggests that gap ligation is polβ and free nucleotide independent and both BER ligases are capable of this aberrant ligation. Lastly, we measured the real-time DNA-binding kinetics of polβ, LIG1 and LIG3α for one nucleotide gap DNA using BLI assay. Our results demonstrated very similar binding affinity with the equilibrium binding constants (*K*_D_) in the range of 3–6 nM for all BER enzymes finalizing the repair pathway at the downstream steps (Supplementary Figure S8).

### Ligation of the nick repair intermediates with 3′-ribonucleotides by LIG1

In the present study, in addition to the coupled assays including polβ, rNTP mismatch, one nucleotide gap DNA and LIG1, we comprehensively investigated the ligation efficiency of LIG1 in the presence of a single ribonucleotide at the 3′-end of nick DNA for all possible 12 ribo-mismatches, *i.e*. 3′-preinserted rA, rG or rC opposite templates A, T, G or C (Supplementary Figure S9A). These nick DNA substrates mimic the ribonucleotide insertion products of DNA polymerases before LIG1 seals the resulting nick intermediate during Okazaki fragment maturation of DNA replication or at the last ligation step of almost all DNA repair pathways.

**Figure 4.**
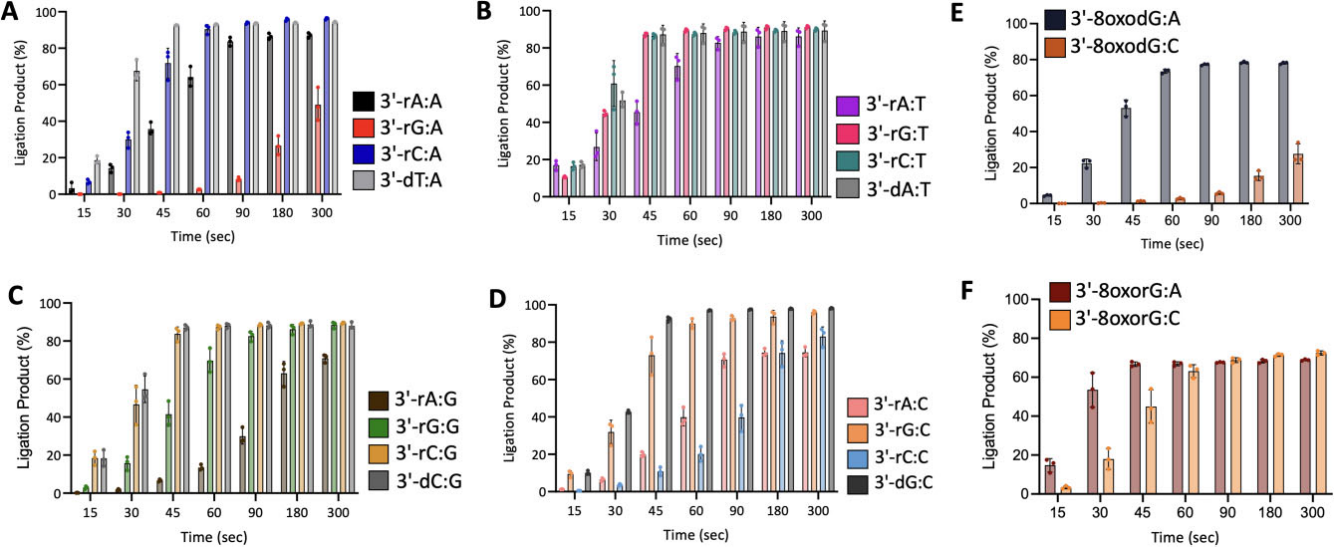
Ligation efficiency of the repair intermediates with 3′-preinserted ribonucleotide mismatches and 8-oxodG versus 8-oxorG. (**A–D**) Graphs show the time-dependent changes in the amount of ligation products for nick DNA substrates containing 3′-preinserted ribonucleotide mismatches opposite template base A, T, G and C. (**E-F**) Graphs show the time-dependent changes in the ligation products for nick DNA substrates containing 3′-preinserted 8-oxodG versus 8-oxorG. The data are presented as the averages from three independent experiments ± SD. Representative gel images showing time-dependent product formation are presented in Supplementary Figures S9–S12.

The ligation of 3′-preinserted ribonucleotide mismatches by LIG1 yielded efficient nick sealing for almost all 12 ribo-mismatches (Figure 4A–D). We observed a robust accumulation of ligation products for the Watson–Crick base paired nick DNA substrates containing 3′-rA:T (Figure 4B and Supplementary Figure S9C, lanes 2–8), 3′-rC:G (Figure 4C and Supplementary Figure S9D, lanes 18–24), and 3′-rG:C (Figure 4D and Supplementary Figure S9E, lanes 10–16). Similarly, LIG1 was able to ligate the repair intermediates containing several 3′-ribonucleotide mismatches as efficiently as canonically base paired ends, 3′-rA:A and 3′-rC:A (Figure 4A and Supplementary Figure S9B, lanes 2–8 and 18–24, respectively), 3′-rG:T and 3′-rC:T (Figure 4B and Supplementary Figure S9C, lanes 10–16 and 18–24, respectively), 3′-rG:G (Figure 4C and Supplementary Figure S9D, lanes 10–16) and 3′-rA:C (Figure 4D and Supplementary Figure S9E, lanes 2–8). However, the ligation efficiency was relatively lower for the nick DNA substrates containing 3′-rA:G (Figure 4C and Supplementary Figure S9D, lanes 2–8) and 3′-rC:C (Figure 4D and Supplementary Figure S9E, lanes 18–24). We obtained the lowest ligation for 3′-rG:A (Figure 4A and Supplementary Figure S9B, lanes 10–16). Overall, our results revealed a time-dependent increase in the amount of nick sealing products with >60% ligation for every 3′-ribo-mismatch except 3′-rG:A (Figure 4).

In control experiments, we confirmed the ligation of the repair intermediates with 3′-preinserted Watson–Crick base pairs containing 3′-dA:T, 3′-dT:A, 3′-dC:G and 3′-dG:C by LIG1 (Supplementary Figure S10). When compared to these control reactions, the results of ribo-mismatch ligations reveal a lack of efficient sugar discrimination by LIG1 for the repair intermediates containing a single ribonucleotide at the 3′-end.

### Ligation of the nick repair intermediates with 3′-mismatches by LIG1

To compare the substrate specificity of LIG1 for nick DNA containing a single deoxyribo- versus ribonucleotide mismatch at the 3′-end, we also performed ligation assays in the presence of all possible 12 non-canonical mismatches, i.e. 3′-preinserted dA, dT, dG or dC opposite templates A, T, G or C.

We obtained subtle differences in the nick sealing of non-canonical 3′-mismatches by LIG1 (Supplementary Figure S11). There were more abundant ligation products over the incubation time for nick DNA substrates containing 3′-dG:T (Supplementary Figure S11B, lanes 10–16), 3′-dC:T (Supplementary Figure S1B, lanes 18–24), and 3′-dT:C (Supplementary Figure S11D, lanes 18–24). We observed simultaneous appearance of DNA-AMP intermediate and nick ligation products for the nick substrates containing 3′-mismatches dA:A (Supplementary Figure S11A, lanes 2–8), dT:T (Supplementary Figure S11B, lanes 2–8), dG:G (Supplementary Figure S11C, lanes 2–8), dT:G (Supplementary Figure S11C, lanes 18–24) and all template base C mismatches (Supplementary Figure S11D). The end-joining ability of LIG1 for the nick substrates containing 3′-dG:A (Supplementary Figure S11A, lanes 10–16) and 3′-dA:G (Supplementary Figure S11C, lanes 10–16) mismatches showed no ligation products at all. Overall, our results demonstrated less efficient nick sealing of all 12 mismatches in comparison with lack of sugar discrimination by LIG1 against the nick DNA substrates containing 3′-ribonucleotides (Figure 4 versus Supplementary Figure S11).

### Ligation of the nick repair intermediates with 3′-8oxodG versu*s* 3′-8oxorG

We next investigated the ligation efficiency of nick DNA substrates containing 3′-preinserted 8-oxodG versus 8-oxorG (Supplementary Figure S12A). Consistent with our previous reports (6,7,9), we observed mutagenic nick sealing of 3′-8oxodG:A, while the ligation of nick DNA substrate containing 3′-8oxodG:C was less efficient resulting in the formation of DNA-AMP intermediate products (Supplementary Figure S12B) and ∼4- to 8-fold difference in the amount of ligation products (Figure 4E). On the other hand, our results demonstrated mutagenic end joining of oxidized ribonucleotide-containing nick DNA substrates 3′-8oxorG:A and 3′-8oxorG:C (Supplementary Figure S12C). We observed a faster accumulation of ligation products for both 3′-8oxorG:A and 3′-8oxorG:C (Figure 4F). Overall, LIG1 does not effectively discriminate against 3′-8oxorG just as it fails to discriminate against all possible 12 ribonucleotide mismatches.

### Removal of 3′-mismatches from the nick repair intermediates by APE1

**Figure 5.**
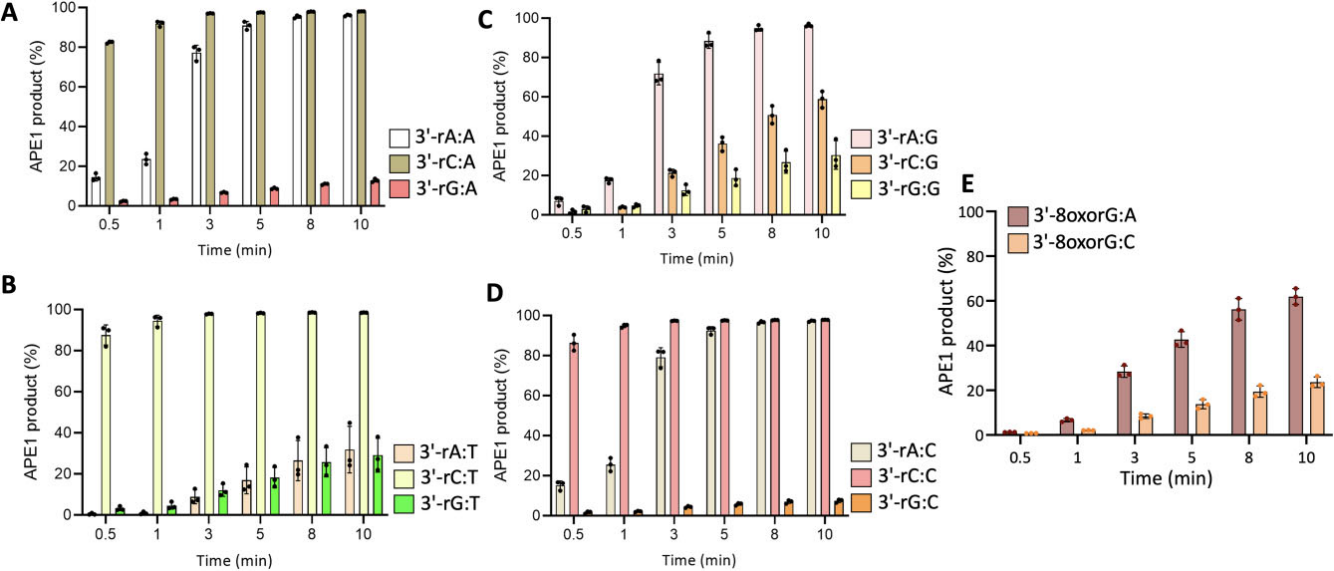
Removal of 3′-ribonucleotide mismatches from the nick repair intermediate by APE1. (**A–E**) Graphs show the time-dependent changes in the amount of removal products of APE1 for 3′-ribonucleotide mismatches from the nick DNA substrates containing template base A, T, G, C and 3′-8-oxorG. The data are presented as the averages from three independent experiments ± SD. The gel images showing the time-dependent substrate and product formation are presented in Supplementary Figure S13.

**Figure 6.**
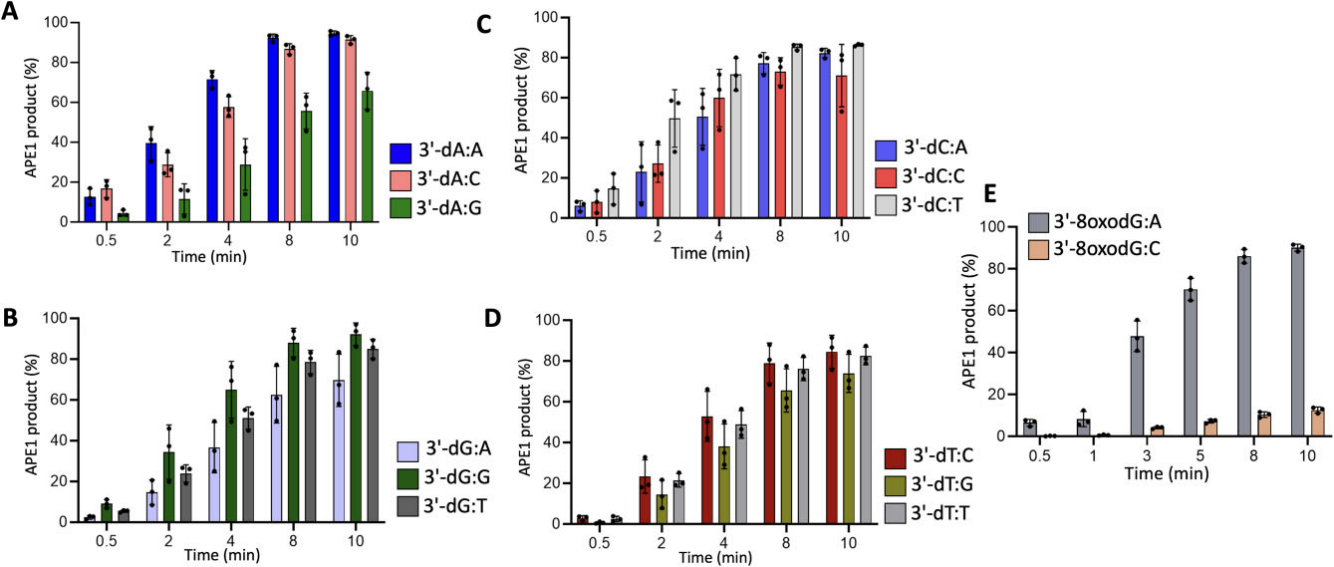
Removal of 3′-mismatches from the nick repair intermediate by APE1. (**A**–**E**) Graphs show the time-dependent changes in the amount of removal products of APE1 for a mismatched base 3′-dA, 3′-dG, 3′-dC, 3′-dT and 3′-8-oxodG from the nick DNA substrates containing template base A, T, G and C. The data are presented as the averages from three independent experiments ± SD. The gel images showing the time-dependent substrate and product formation are presented in Supplementary Figure S14.

In addition to its endonuclease function, using the same rigid active site, APE1 has been shown to exhibit 3′-5′-exonuclease activity to remove mismatches and various forms of oxidative damage incorporated by polβ during BER (8,38,39). Yet, it is unclear whether APE1 could serve as a proofreader of ribonucleotide-containing repair intermediates that could be formed due to aberrant BER pathway coordination at the final steps. In the present study, we comprehensively investigated the substrate specificity of APE1 exonuclease activity for nick DNA substrates containing all 12 possible mismatches, ribonucleotides, and oxidized bases at the 3′-end (Figures 5-6).

For the nick DNA substrates containing ribonucleotide mismatches, our results demonstrated that APE1 shows distinct efficiency depending on the hydrogen bonding characteristics of the terminal base pair and the identity of the 3′-ribonucleotide (Figure 5 and Supplementary Figure S13). We observed poor efficiency in removing ribonucleotides that were Watson–Crick base paired to their complementary DNA base: 3′-rA:T, 3′-rC:G and 3′-rG:C (Figure 5B,D). The highest amount of ribonucleotide removal products were obtained for the nick DNA substrates containing 3′-rA:A, 3′-rA:G, 3′-rA:C, 3′-rC:A, 3′-rC:T and 3′-rC:C mismatches (Figure 5A,D). Interestingly, APE1 shows poor efficiency for the removal of 3′-rG opposite template A, T and G.

We then extended the analysis of APE1 exonuclease activity for the repair intermediates containing all possible 3′-preinserted 12 non-canonical mismatches (Figure 6 and Supplementary Figure S14). Our results demonstrated that APE1 exhibits no significant differences for the purine:pyrimidine, pyrimidine:purine or pyrimidine:pyrimidine mispairs. There was a variability with purine:purine mismatches such as 3′-dA:A, which shows the most efficient mismatch removal out of all tested nick DNA substrates (Figure 6A). Furthermore, we observed the lowest mismatch excision for purine:purine mispair substrates of differing purines 3′-dG:A and 3′-dA:G (Figure 6A,C). As expected, APE1 was relatively less efficient for the removal of 3′-bases from the nick DNA substrates containing Watson–Crick canonical ends (Supplementary Figure S15). Lastly, we compared the efficiency of 3′-8oxodG versus 3′-8oxorG removal by APE1 (Figures 5E and 6E). Our results showed that APE1 can remove 3′-8oxodG or 3′-8oxorG damaged bases from the nick DNA substrates containing template base A more efficiently than template C (Supplementary Figure S16).

Our overall results revealed that APE1 shows distinct efficiencies for the removal of mismatched bases depending on the 3′-end:template base pair architecture of the nick repair intermediate. It can also proofread nicks containing a damaged base depending on the strength of base paring interaction with the template. However, for almost almost all nick DNA substrates with a single ribonucleotide at 3′-end, excluding those with 3′-rG, our results demonstrate that APE1 is capable of efficiently proofreading ribonucleotide mismatches.

## Discussion

BER involves the coordinated channeling of repair intermediates from one enzyme to the next in the repair pathway, which prevents the accumulation of potentially toxic strand-break intermediates in cells. While it is generally assumed that DNA repair operates to preserve genome integrity, this mechanism is not always precise. Evidence, particularly from our studies, is emerging that BER can contribute to genome instability if normal coordination breaks down (6–9). Inaccurate BER, stemming from deviations in the proper repair pathway coordination from polβ gap filling to DNA ligase nick sealing at the downstream steps, may result in an accumulation of aberrant repair intermediates which could lead to cellular toxicity and genome instability. The one nucleotide gap formed after APE1-mediated strand incision, which requires 5′-end processing, gap filling synthesis and subsequent nick sealing, could be a particularly deleterious intermediate that interferes with the completion of accurate repair. Failures in the DNA synthesis step by polβ, could lead to persistent one nucleotide gaps if left unfilled, which is expected to be a deleterious repair intermediate (17,40). In the present study, we investigated how LIG1 responds at the last nick sealing step if polβ is unable to fill the one nucleotide gap during the prior DNA synthesis step of the BER pathway. Our results demonstrate that aberrant gap filling by polβ results in ligation of the one nucleotide gap itself, which is especially mutagenic as it leads to a single-nucleotide deletion product.

Our first observation was that unfilled gaps by polβ are ligated by LIG1 in the presence of ribonucleotides, which exist several orders of magnitude higher than deoxyribonucleotides within cells, especially within terminally differentiated cells, where the discrepancy can be even higher (11–14). Polβ is an error prone polymerase belonging to the X-family of DNA polymerases which utilize a backbone carbonyl to clash with the 2′-OH of rNTPs (31–33,41–43). Previous work has highlighted that this strategy results in a polymerase with a steric ‘fence’ rather than the steric ‘gates’ of A- and B-family of polymerases (32,33) Due to the lower sugar discrimination of polβ, as compared to replicative polymerases, as well as the abundance of rNTPs, it has previously been estimated that polβ inserts a rNTP every 81 insertion events under physiological conditions (32). However, under our assay conditions, we did not detect rNTP incorporation nor ligation of ribonucleotide incorporation products. Upon mutation of the steric gate residue, we observed ribonucleotide incorporation products and nick ligation products for rGTP:C and 8oxorGTP:A insertion and coupled reactions, respectively, indicating that the Y271A mutation allows for insertion of Watson–Crick or Hoogsteen base pairs.

We also observed defective gap filling by the polβ cancer-associated variants Y265C, E288K and E295K. We found that reduction or ablation of gap filling by these polβ mutants results in the accumulation of gap ligation products by LIG1. These results indicate that other polβ cancer-associated variants with known aberrant BER activity, including R152C, S229L, G231D and I260M, may also result in defective gap filling and accumulation of single-nucleotide deletion mutagenesis products (17,30,44–50). We also note that since more gap ligation products were observed for gap DNA containing template base C over all other template bases, it is possible that cancer patients with these mutations might preferentially accumulate dG deletions from the genome in response to defective BER stemming from unfilled gaps by polβ. Future structural studies of LIG1 in complex with gap DNA will be needed to explain the template C preference of LIG1. We hypothesize that in order to bring the 3′-OH and 5′-P of the gap close enough to react, LIG1 could either be looping out the templating base or locally melting the upstream strand. It is known that LIG1 enforces an underwound conformation upstream of the nick, so perhaps this flexibility allows close positioning of the reactive groups (51). If a specific interaction is made between the looped out templating nucleotide and the LIG1 active site, this mechanism could potentially explain the apparent substrate specificity of gap ligation.

Additionally, we demonstrated that gap ligation of the one nucleotide gap is polβ and free nucleotide independent so it is possible that in other scenarios where one nucleotide gaps are found, if the gap is not filled efficiently by repair or replication polymerase, LIG1 may attempt to ligate it. Furthermore, gap ligation is not specific to LIG1, as other non-human ligases, such as T4 DNA ligase, have been previously shown to ligate one nucleotide gaps as well (52).

**Figure 7.**
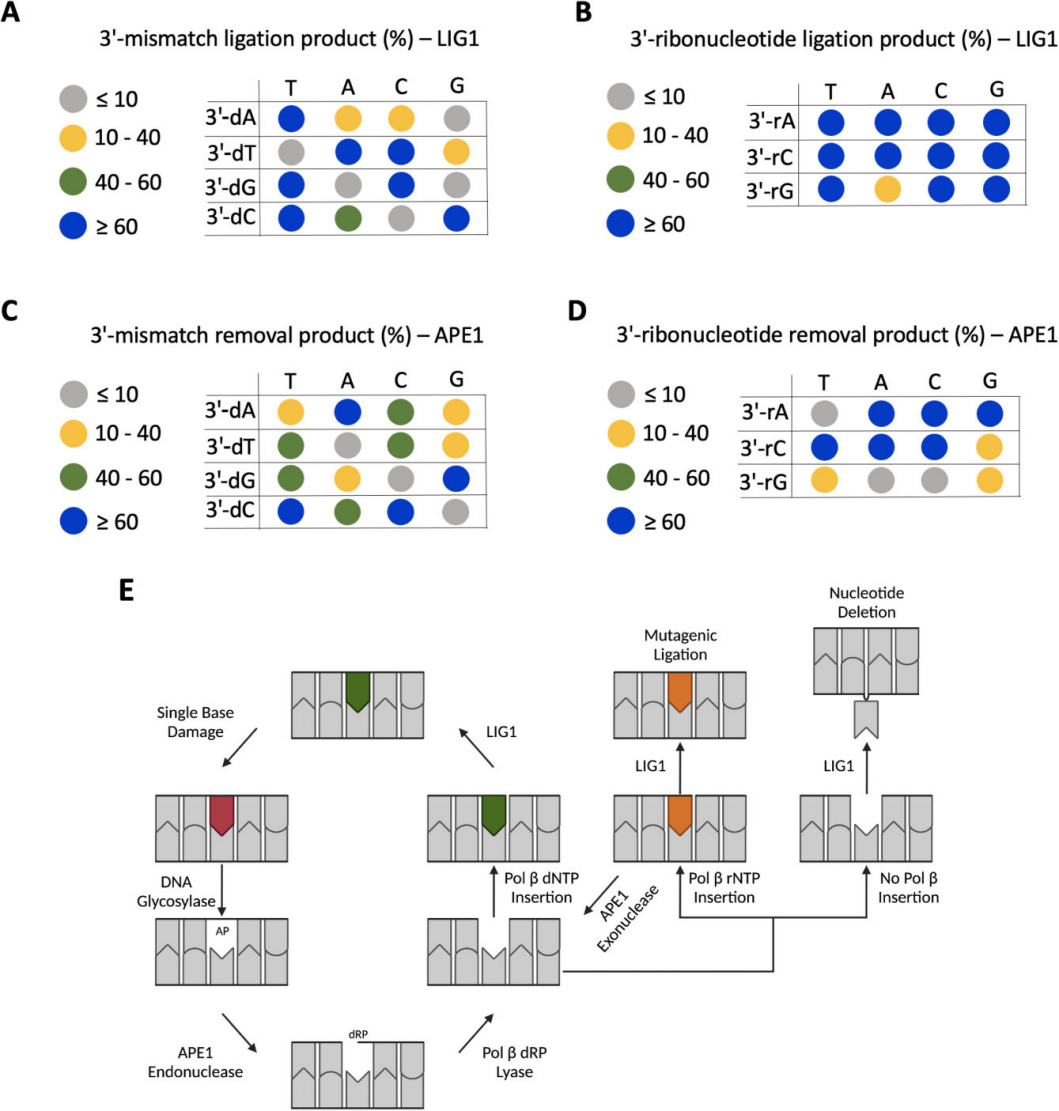
Efficiencies of APE1 and LIG1 for proofreading versus nick sealing of nick repair intermediates with 3′-deoxyribonucleotide and ribonucleotide mismatches. (**A-B**) Dot plots show LIG1 efficiency, represented by the colors indicated in the figure, for the ligation of the nick DNA substrates with 3′-deoxyribonucleotide (A) and 3′-ribonucleotide (B) mismatches for 3 min. (**C-D**) Dot plots show the APE1 efficiency, represented by the colors indicated in the figure, for the exonuclease removal of a base from the nick DNA substrates with 3′-deoxyribonucleotide for 4 min (C) and 3′-ribonucleotide for 3 min (D) mismatches. (**E**) The working model showing how the deviations in the substrate-product channeling process in the presence of ribonucleotides impact the efficiency of BER at the downstream steps of the repair pathway.

In the case of ribonucleotide incorporation by repair or replicative DNA polymerases, the resulting nick product can serve as a substrate for LIG1 to seal at the last ligation step. In the present study, we observed that LIG1 is capable of efficient ligation of almost all ribonucleotide-containing mismatches at the 3′-end of the nick, which is in stark contrast to the efficiency of deoxyribonucleotide-containing mismatches (Figure 7A,B). This demonstrates a complete lack of sugar discrimination by LIG1 and highlights the importance of polβ for excluding ribonucleotides during BER, as any incorporated ribonucleotides would be readily ligated by LIG1. Mechanistically, it is known that ligases enforce a 2′-endo to 3′-endo sugar pucker conformational change in the 3′-nucleotide which is critical for catalysis; therefore, it is possible that the reason 3′-ribonucleotides are efficiently ligated is because ribonucleotides naturally prefer the 3′-endo conformation, which allows close-to-optimal positioning of reactive groups, even in the absence of proper base pairing between the 3′-nick terminus (8,51,53). However, future structure/function studies of LIG1 in complex with nick DNA containing 3′-ribonucleotides will be critical to confirm this hypothesis.

APE1 has previously been shown to serve as a compensatory proofreading enzyme for polβ-mediated errors introduced during BER (8,38,39). Our results revealed for the first time that APE1 is capable of the removal of a single 3′-ribonucleotide from the nick repair intermediates, particularly 3′-rA and 3′-rC while it was less efficient on 3′-rG containing nicks. This suggests that inserted 3′-rG might escape proofreading by APE1 and be incorporated into the genome more frequently, especially due to the highly efficient ligation of 3′-rG:C and 3′-rG:T. Furthermore, we observed that APE1 was capable of removing 3′-8oxorG with moderate efficiency opposite template A and C, suggesting that APE1 serves as a potential line of defense against the incorporation of this highly mutagenic lesion into the genome. Additionally, APE1 shows different efficiency for the removal of 3′-mismatched base depending on the architecture of the 3′-end; however, all 12 mismatches were capable of removal by APE1, demonstrating the broad range of suitable substrates for APE1 exonuclease activity (Figure 7C,D). From these results, as well as our previous study showing physical and function interaction of APE1/LIG1, we suggest that APE1 plays an unappreciated role as a vital proofreader of polβ to prevent mutagenic repair, further supporting the notion that the multi-protein BER complex, including APE1, polβ and LIG1, is required for faithful BER (8). Without the exonuclease activity of APE1, errors introduced by polβ would be more likely to be incorporated into the genome, leading to greater BER-induced genome instability.

In light of the insights gained from this study, we propose the following working model for how unfilled gaps left by polβ could affect substrate-product channeling at the downstream steps of BER (Figure 7E). As the main BER DNA polymerase, polβ discriminates against rNTPs and does not insert them often, but when polβ fails to insert any nucleotide, whether due to ribonucleotide challenge or disease-associated mutation, LIG1 may attempt to ligate the one nucleotide gap which results in a single-nucleotide deletion. This single-nucleotide deletion may be even more deleterious then an embedded ribonucleotide, since at least the ribonucleotide still retains base coding potential. Therefore, polβ has a limited amount of time to incorporate nucleotides from the nucleotide pool before LIG1 ligates the one nucleotide gap. When polβ inserts a rNTP, it is swiftly channeled to LIG1 which ligates the nick, finalizing incorporation of the inserted ribonucleotide into genomic DNA. Therefore, the ribonucleotide discrimination of polβ is the only line of defense for preventing BER-mediated ribonucleotide incorporation. Instances where polβ has greater ribonucleotide tolerance, or when the gap filling activity of polβ is substituted by a polymerase with greater ribonucleotide tolerance, will likely result in increased BER-mediated ribonucleotide incorporation. However, as APE1 can efficiently remove the majority of 3′-ribonucleotides from a nick DNA substrate, it is possible that in the multiprotein BER complex, channeling between polβ ribonucleotide insertion and LIG1 ligation is interrupted by the proofreading activity of APE1, thereby preventing mutagenic incorporation and subsequent ligation at the downstream steps. Additionally, in disease states where the gap filling activity of polβ is compromised, any other X-family repair DNA polymerase, such as polλ or polμ, may take over to prevent the mutagenic consequences of unfilled gaps (54,55). Future experiments will be directed at understanding how the multi-protein BER complex consisting of APE1, polβ and LIG1 function together to ensure accurate repair by excluding ribonucleotides and preventing unfilled gaps. Defining the molecular determinants that dictate BER accuracy, particularly in the context of pathway coordination, is critical to fully understand disease mechanisms and how defects in this system contribute to disease risk.

## Supplementary Material

gkae104_Supplemental_File

## Data Availability

The data underlying this article will be shared on reasonable request to the corresponding author.
