# Supplementary material for: Unfilled gaps by polβ lead to aberrant ligation by LIG1 at the downstream steps of base excision repair pathway
Source: Nucleic Acids Res. 2024 Feb 16;52(7):3810–22. doi: 10.1093/nar/gkae104 (PMC11039997; doi:10.1093/nar/gkae104)
Supplement: gkae104_Supplemental_File [file gkae104_supplemental_file.pdf]

Unfilled gaps by polβ leads to aberrant ligation by LIG1 at the downstream steps of base excision repair pathway

Mitchell Gulkis, Ernesto Martinez, Danah Almohdar, Melike Çağlayan*

Department of Biochemistry and Molecular Biology, University of Florida, Gainesville, FL 32610, USA

*To whom correspondence should be addressed. Tel.: +1 352-294-8383; Email: caglayanm@ufl.edu

Supplementary Information

Supplementary Figures 1-16

Supplementary Tables 1-6

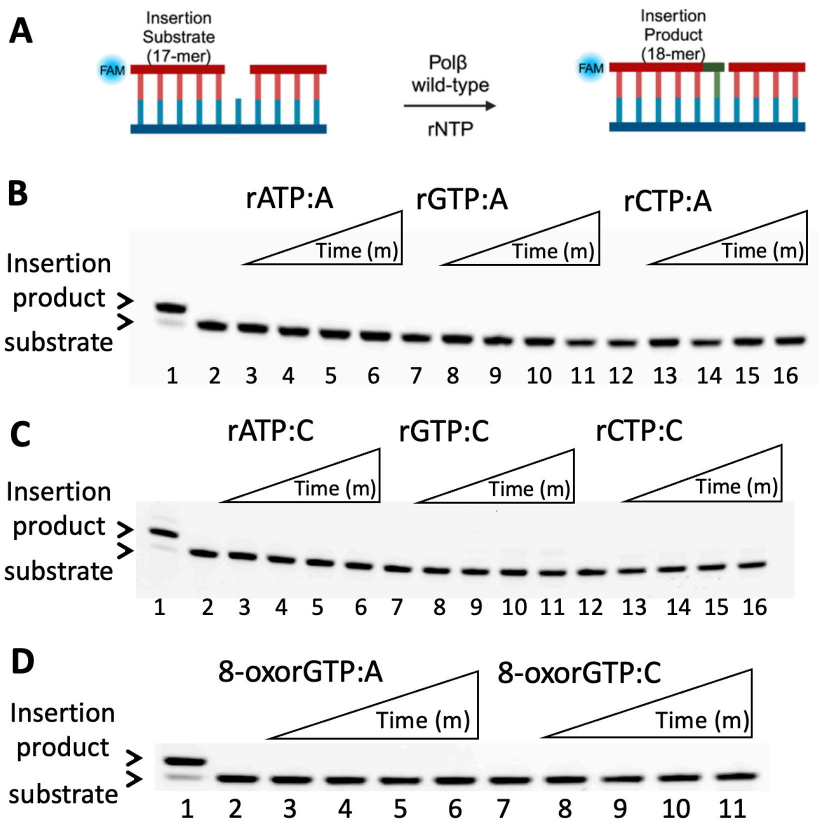

Supplementary Figure 1. Ribonucleotide mismatch insertions by polβ. (A) Scheme showing the substrate and product observed in the insertion assay. **(B-C)** Line 1 is the positive control showing dTTP:A (B) and dGTP:C (C) insertion products by polβ. Lanes 2, 7, and 12 are the negative enzyme controls of gap DNA substrates containing template base A and C, respectively. Lanes 3-6, 8-11, and 13-16 are the ribonucleotide mismatch insertion products by polβ, and correspond to time points of 0.5, 1, 3, and 5 min. **(D)** Line 1 is the positive control showing dGTP:C insertion product by polβ. Lanes 2 and 7 are the negative enzyme controls of gap DNA substrates containing template base A and C, respectively. Lanes 3-6 and 8-11 are 8-oxorGTP insertion products by polβ, and correspond to time points of 0.5, 1, 3, and 5 min. Representative gel images of three independent repeats.

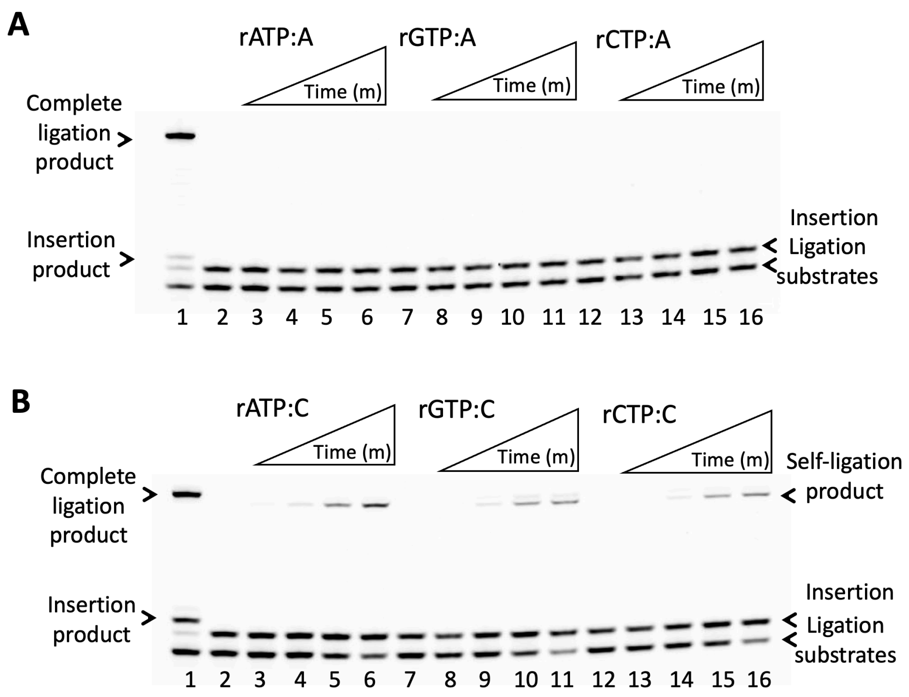

Supplementary Figure 2. Ligation efficiency of polβ ribonucleotide insertions by LIG3α. (A-B) Line 1 is the positive control showing the ligation of polβ dTTP:A and dGTP:C insertion products by LIG3α. Lanes 2, 7, and 12 are the negative enzyme controls of gap DNA substrates containing template base A or C. Lanes 3-6, 8-11, and 13-16 are the reaction products in the presence of polβ, LIG3α, and rNTP as indicated in the figure, and correspond to time points of 0.5, 1, 3, and 5 min. Representative gel images of three independent repeats.

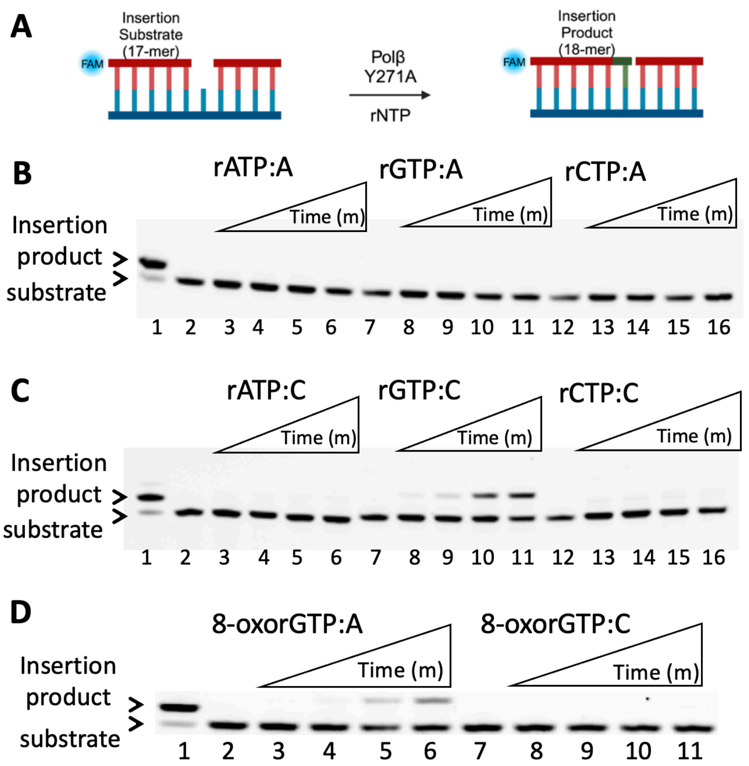

Supplementary Figure 3. Ribonucleotide mismatch insertions by polβ Y271A mutant. (A) Scheme showing the substrate and product observed in the insertion assay. **(B-C)** Line 1 is the positive control showing dTTP:A and dGTP:C insertion products by polβ Y271A mutant. Lanes 2, 7, and 12 are the negative enzyme controls of gap DNA substrates containing template base A and C, respectively. Lanes 3-6, 8-11, and 13-16 are the ribonucleotide mismatch insertion products by polβ Y271A mutant, and correspond to time points of 0.5, 1, 3, and 5 min. **(D)** Line 1 is the positive control showing dGTP:C insertion product by polβ Y271A mutant. Lanes 2 and 7 are the negative enzyme controls of gap DNA substrates containing template base A and C, respectively. Lanes 3-6 and 8-11 are 8-oxorGTP insertion products by polβ Y271A mutant, and correspond to time points of 0.5, 1, 3, and 5 min. Representative gel images of three independent repeats.

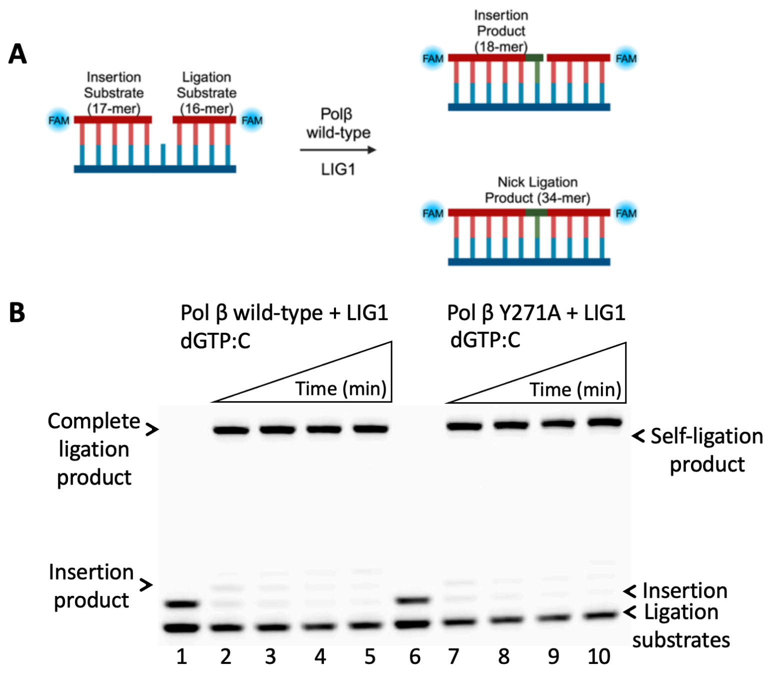

Supplementary Figure 4. Ligation of polβ correct nucleotide insertion products by LIG1. (A) Scheme showing the substrates and products observed in the coupled assay. **(B)** Lane 1 and 6 are the negative enzyme controls of gap DNA substrate containing template C. Lanes 2-5 and 7-10 are the ligation of dGTP:C insertion products in the coupled reaction products in the presence of polβ wild-type and Y271A mutant, respectively, and correspond to time points of 0.5, 1, 3, and 5 min. Representative gel images of three independent repeats.

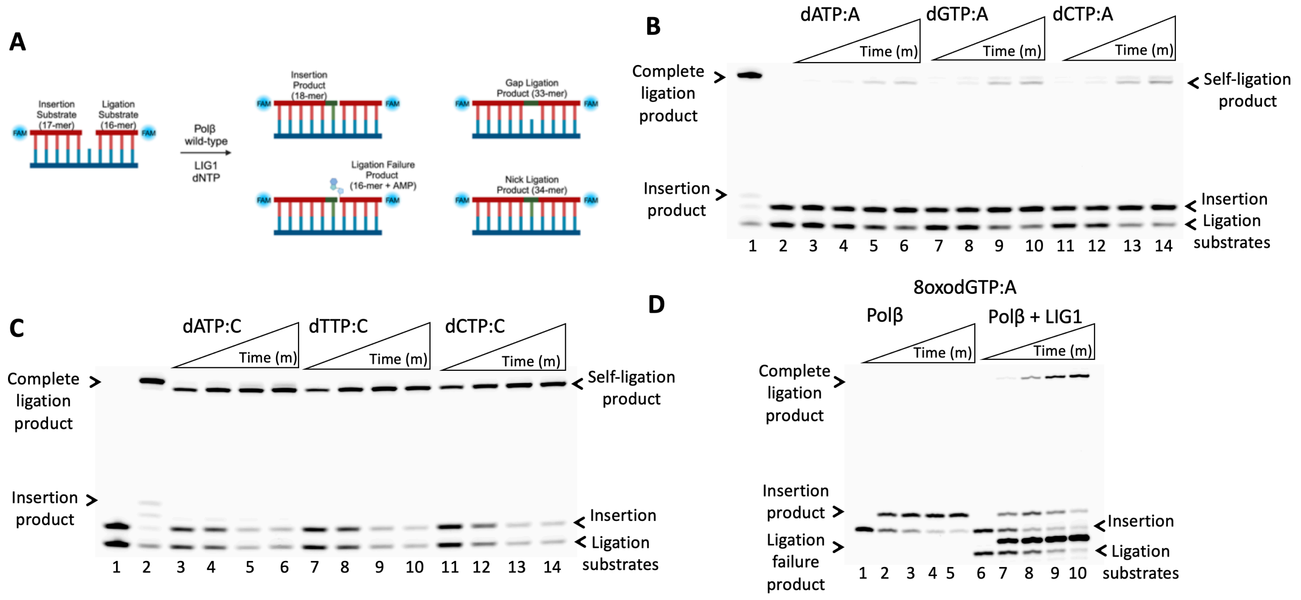

Supplementary Figure 5. Ligation efficiency of polβ mismatch insertions by LIG1. (A) Scheme showing the substrates and products observed in the coupled assay. **(B-C)** Line 1 is the positive control showing the ligation of polβ dTTP:A and dGTP:C insertion products by LIG1. Line 2 is the negative enzyme control of gap DNA substrates containing template base A or C. Lanes 3-6, 7-10, and 11-14 are the coupled reaction products in the presence of polβ, LIG1, and the dNTP as indicated in the figure, and correspond to time points of 0.5, 1, 3, and 5 min. Representative gel images of three independent repeats. **(D)** Lanes 1 and 6 are the negative enzyme controls for 8oxodGTP:A insertion and coupled reactions, respectively. Lanes 2-5 are the insertion reaction products in the presence of polβ, 8oxodGTP, and one nucleotide gap DNA with template A, and correspond to time points of 0.5, 1, 3, and 5 min. Lanes 7-10 are the coupled reaction products in the presence of polβ, LIG1, 8oxodGTP, and one nucleotide gap DNA with template A, and correspond to time points of 0.5, 1, 3, and 5 min. Representative gel images of three independent repeats.

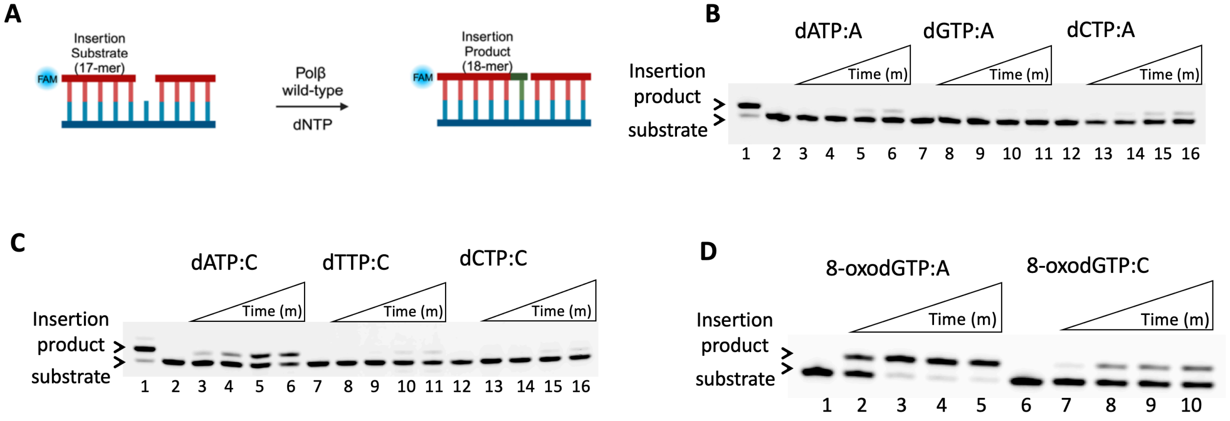

Supplementary Figure 6. Mismatch nucleotide insertions by polβ. (A) Scheme showing the substrate and product observed in the insertion assay. **(B-C)** Line 1 is the positive control showing dTTP:A and dGTP:C insertion products by polβ. Lanes 2, 7, and 12 are the negative enzyme controls of gap DNA substrates containing template base Aand C, respectively. Lanes 3-6, 8-11, and 13-16 are the mismatch insertion products by polβ, and correspond to time points of 0.5, 1, 3, and 5 min. **(D)** Lanes 1 and 6 are the negative enzyme controls of gap DNA substrates containing template base A and C, respectively. Lanes 2-5 and 7-10 are 8-oxodGTP insertion products by polβ, and correspond to time points of 0.5, 1, 3, and 5 min. Representative gel images of three independent repeats.

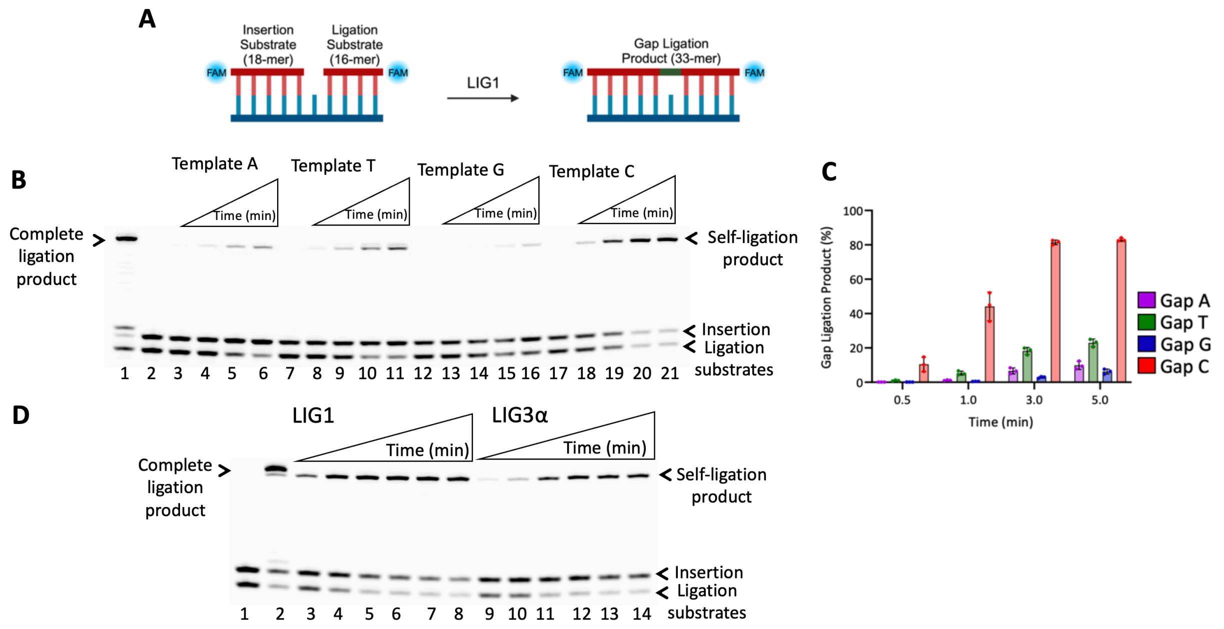

Supplementary Figure 7. Ligation of one nucleotide gap DNA substrate by LIG1 and LIG3α. **(A)** Scheme showing the substrate and product observed in the gap ligation assay. **(B)** Line 1 is the ligation of polβ dTTP:A insertion product by LIG1. Lanes 2, 7, 12, and 17 are the negative enzyme controls of gap DNA substrates containing template base A, T, G, and C, respectively. Lanes 3-6, 8-11, 13-16, and 18-21 are the ligation products of the one nucleotide gap substrate with template base A, T, G, and C respectively, and correspond to time points of 0.5, 1, 3, and 5 min. **(C)** Graph shows the time-dependent changes in the amount of gap ligation products, and the data are presented as the averages from three independent experiments ± SDs. **(D)** Line 1 is the negative enzyme control of gap DNA substrate containing template base C and line 2 is the ligation of polβ dGTP:C insertion product by LIG3α. Lanes 3-8 and 9-14 are the ligation products of the one nucleotide gap substrate with template base C by LIG1 and LIG3α, respectively, and correspond to time points of 0.5, 1, 3, 5, 8, and 10 min.

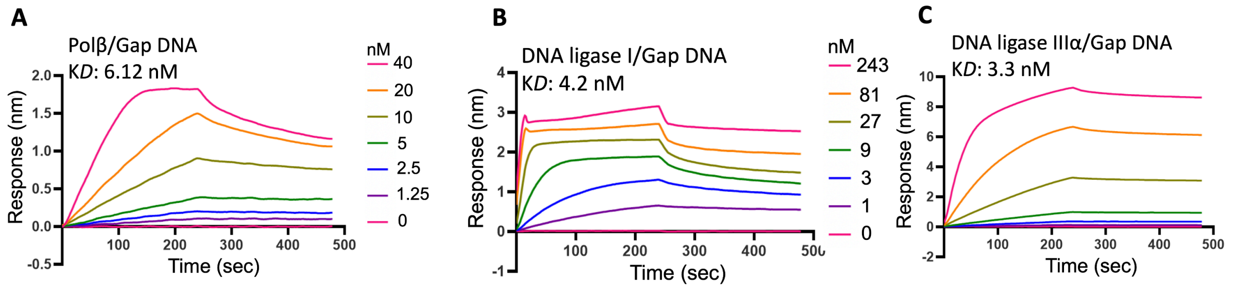

Supplementary Figure 8. DNA binding kinetics of polβ, LIG1, and LIG3α. Real-time gap DNA binding kinetics and the equilibrium binding constants (K_D) are shown for polβ (**A**), LIG1 (**B**), and LIG3α (**C**). Sensorgrams are shown for the concentrations range of BER proteins where the DNA molecule with a biotin label is immobilized on the streptavidin biosensors. The data are processed and analyzed with the ForteBio Data Analysis software with 1:1 binding model.

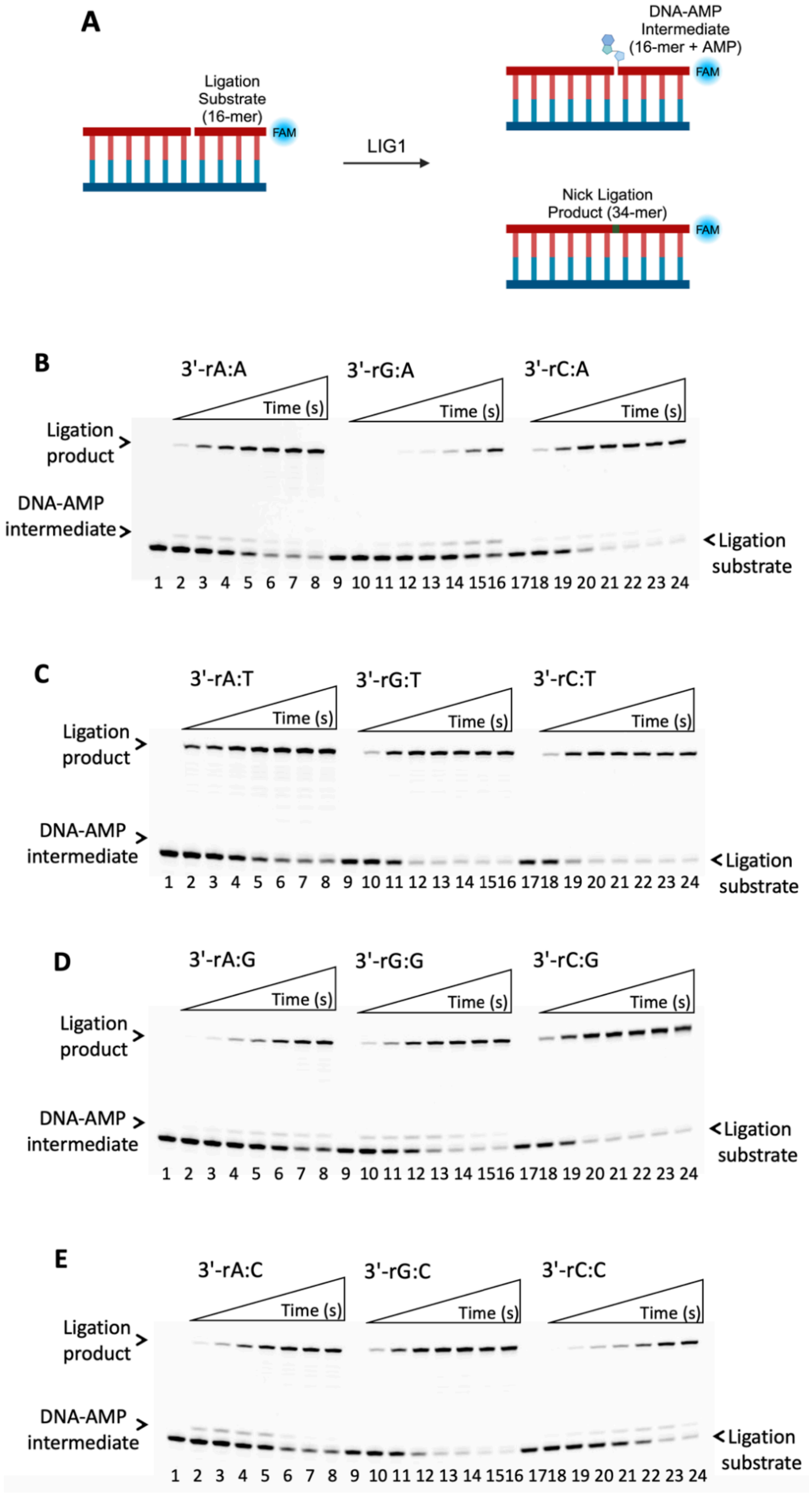
A
Ligation Substrate (16-mer)
FAM
LIG1
DNA-AMP Intermediate (16-mer + AMP)
FAM
Nick Ligation Product (34-mer)
FAM
B
3'-rA:A
3'-rG:A
3'-rC:A
Time (s)
Ligation product
DNA-AMP intermediate
Ligation substrate
1 2 3 4 5 6 7 8 9 10 11 12 13 14 15 16 17 18 19 20 21 22 23 24
C
3'-rA:T
3'-rG:T
3'-rC:T
Time (s)
Ligation product
DNA-AMP intermediate
Ligation substrate
1 2 3 4 5 6 7 8 9 10 11 12 13 14 15 16 17 18 19 20 21 22 23 24
D
3'-rA:G
3'-rG:G
3'-rC:G
Time (s)
Ligation product
DNA-AMP intermediate
Ligation substrate
1 2 3 4 5 6 7 8 9 10 11 12 13 14 15 16 17 18 19 20 21 22 23 24
E
3'-rA:C
3'-rG:C
3'-rC:C
Time (s)
Ligation product
DNA-AMP intermediate
Ligation substrate
1 2 3 4 5 6 7 8 9 10 11 12 13 14 15 16 17 18 19 20 21 22 23 24

Supplementary Figure 9. Ligation of repair intermediates with 3'-preinserted ribonucleotide mismatches. (A) Scheme showing the substrate and products observed in the ligation assay. **(B)** Lanes 1, 9, and 17 are the negative enzyme controls of the nick DNA substrates with template A. Lanes 2-8, 10-16, and 18-24 are the ligation reaction in the presence of 3'-rA:A, 3'-rG:A, and 3'-rC:A, and correspond to time points of 15, 30, 45, 60, 90, 180, and 300 sec. Representative gel image of three independent repeats. **(C)** Lanes 1, 9, and 17 are the negative enzyme controls of the nick DNA substrates with template T. Lanes 2-8, 10-16, and 18-24 are the ligation reaction in the presence of 3'-rA:T, 3'-rG:T, and 3'-rC:T and correspond to time points of 15, 30, 45, 60, 90, 180, and 300 sec. Representative gel image of three independent repeats. **(D)** Lanes 1, 9, and 17 are the negative enzyme controls of the nick DNA substrates with template G. Lanes 2-8, 10-16, and 18-24 are the ligation reaction in the presence of 3'-rA:G, 3'-rG:G, and 3'-rC:G, and correspond to time points of 15, 30, 45, 60, 90, 180, and 300 sec. Representative gel image of three independent repeats. **(E)** Lanes 1, 9, and 17 are the negative enzyme controls of the nick DNA substrates with template C. Lanes 2-8, 10-16, and 18-24 are the ligation reaction in the presence of 3'-rA:C, 3'-rG:C, and 3'-rC:C and correspond to time points of 15, 30, 45, 60, 90, 180, and 300 sec. Representative gel image of three independent repeats. Graphs showing the time-dependent changes in the amount of ligation products of LIG1 are presented in Figure 4.

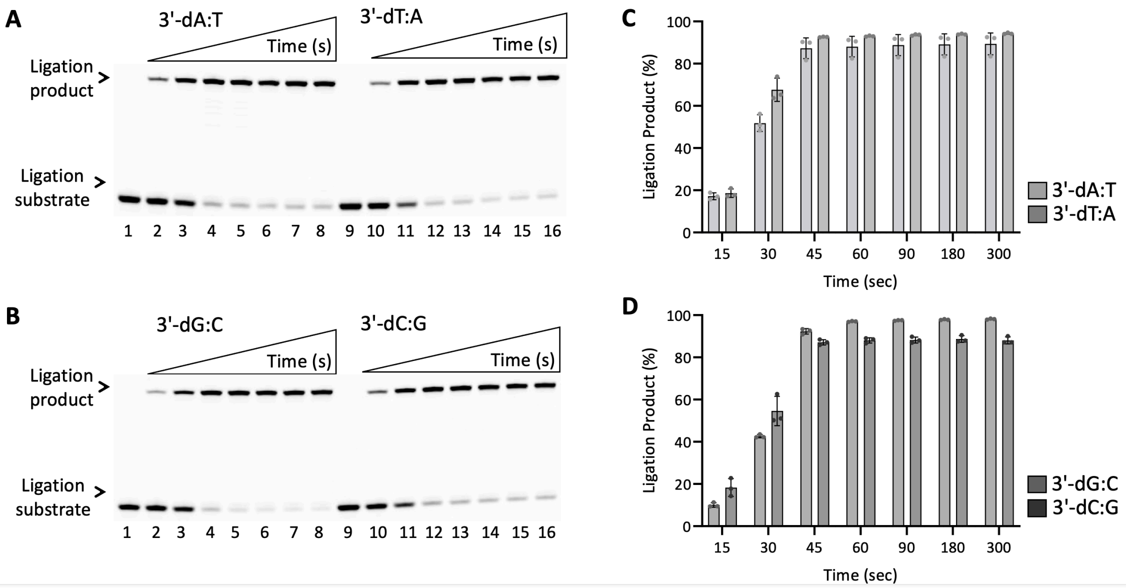

Supplementary Figure 10. Ligation of nick DNA substrate with 3'-preinserted Watson-Crick base paired ends. (A) Lanes 1 and 9 are the negative enzyme controls of the nick DNA substrates with 3'-dA:T and 3'-dT:A, respectively. Lanes 2-8 and 10-16 are the ligation products in the presence of 3'-dA:T and 3'-dT:A, and correspond to time points of 15, 30, 45, 60, 90, 180, and 300 sec. **(B)** Lanes 1 and 9 are the negative enzyme controls of the nick DNA substrates with 3'-dG:C and 3'-dC:G, respectively. Lanes 2-8 and 10-16 are the ligation products in the presence of 3'-dG:C and 3'-dC:G, and correspond to time points of 15, 30, 45, 60, 90, 180, and 300 sec. **(C-D)** Graphs show the time-dependent changes in the amount of ligation products, and the data are presented as the averages from three independent experiments ± SDs.

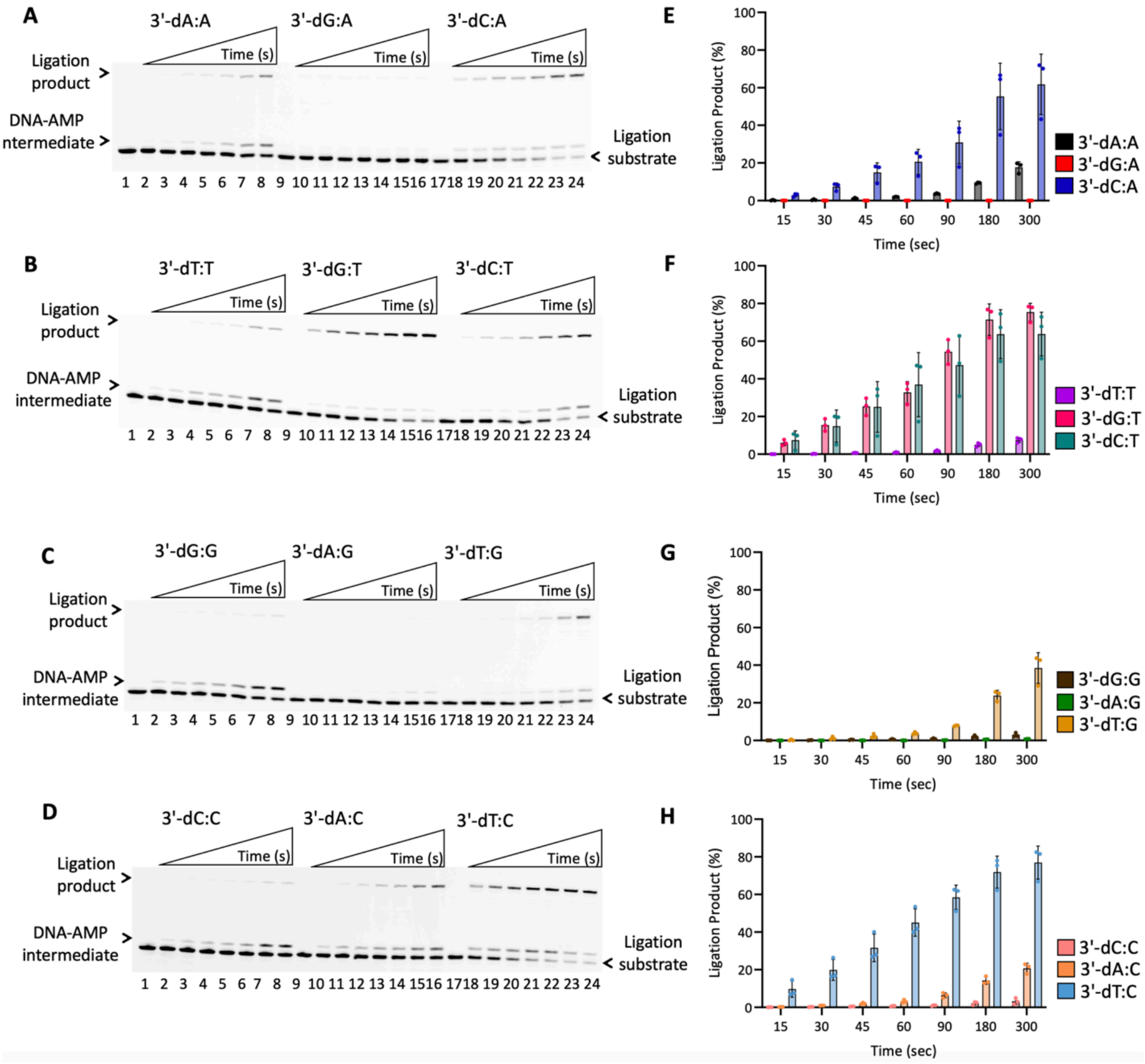

A
3'-dA:A
3'-dG:A
3'-dC:A
Time (s)
Ligation product
DNA-AMP ntermediate
Ligation substrate
1 2 3 4 5 6 7 8 9 10 11 12 13 14 15 16 17 18 19 20 21 22 23 24
B
3'-dT:T
3'-dG:T
3'-dC:T
Time (s)
Ligation product
DNA-AMP intermediate
Ligation substrate
1 2 3 4 5 6 7 8 9 10 11 12 13 14 15 16 17 18 19 20 21 22 23 24
C
3'-dG:G
3'-dA:G
3'-dT:G
Time (s)
Ligation product
DNA-AMP intermediate
Ligation substrate
1 2 3 4 5 6 7 8 9 10 11 12 13 14 15 16 17 18 19 20 21 22 23 24
D
3'-dC:C
3'-dA:C
3'-dT:C
Time (s)
Ligation product
DNA-AMP intermediate
Ligation substrate
1 2 3 4 5 6 7 8 9 10 11 12 13 14 15 16 17 18 19 20 21 22 23 24
E
Ligation Product (%)
0 20 40 60 80 100
15 30 45 60 90 180 300
Time (sec)
3'-dA:A
3'-dG:A
3'-dC:A
F
Ligation Product (%)
0 20 40 60 80 100
15 30 45 60 90 180 300
Time (sec)
3'-dT:T
3'-dG:T
3'-dC:T
G
Ligation Product (%)
0 20 40 60 80 100
15 30 45 60 90 180 300
Time (sec)
3'-dG:G
3'-dA:G
3'-dT:G
H
Ligation Product (%)
0 20 40 60 80 100
15 30 45 60 90 180 300
Time (sec)
3'-dC:C
3'-dA:C
3'-dT:C

Supplementary Figure 11. Ligation efficiency of the repair intermediates with 3'-preinserted mismatches. (A) Lanes 1, 9, and 17 are the negative enzyme controls of the nick DNA substrates with template A. Lanes 2-8, 10-16, and 18-24 are the ligation reaction in the presence of 3'-dA:A, 3'-dG:A, and 3'-dC:A, and correspond to time points of 15, 30, 45, 60, 90, 180, and 300 sec. Representative gel image of three independent repeats. **(B)** Lanes 1, 9, and 17 are the negative enzyme controls of the nick DNA substrates with template T. Lanes 2-8, 10-16, and 18-24 are the ligation reaction in the presence of 3'-dT:T, 3'-dG:T, and 3'-dC:T and correspond to time points of 15, 30, 45, 60, 90, 180, and 300 sec. Representative gel image of three independent repeats. **(C)** Lanes 1, 9, and 17 are the negative enzyme controls of the nick DNA substrates with template G. Lanes 2-8, 10-16, and 18-24 are the ligation reaction in the presence of 3'-dG:G, 3'-dA:G, and 3'-dT:G, and correspond to time points of 15, 30, 45, 60, 90, 180, and 300 sec. Representative gel image of three independent repeats. **(D)** Lanes 1, 9, and 17 are the negative enzyme controls of the nick DNA substrates with template C. Lanes 2-8, 10-16, and 18-24 are the ligation reaction in the presence of 3'-dC:C, 3'-dA:C, and 3'-dT:C and correspond to time points of 15, 30, 45, 60, 90, 180, and 300 sec. Representative gel image of three independent repeats. **(E-H)** Graphs show the time-dependent changes in the amount of ligation products, the data are presented as the averages from three independent experiments ± SD.

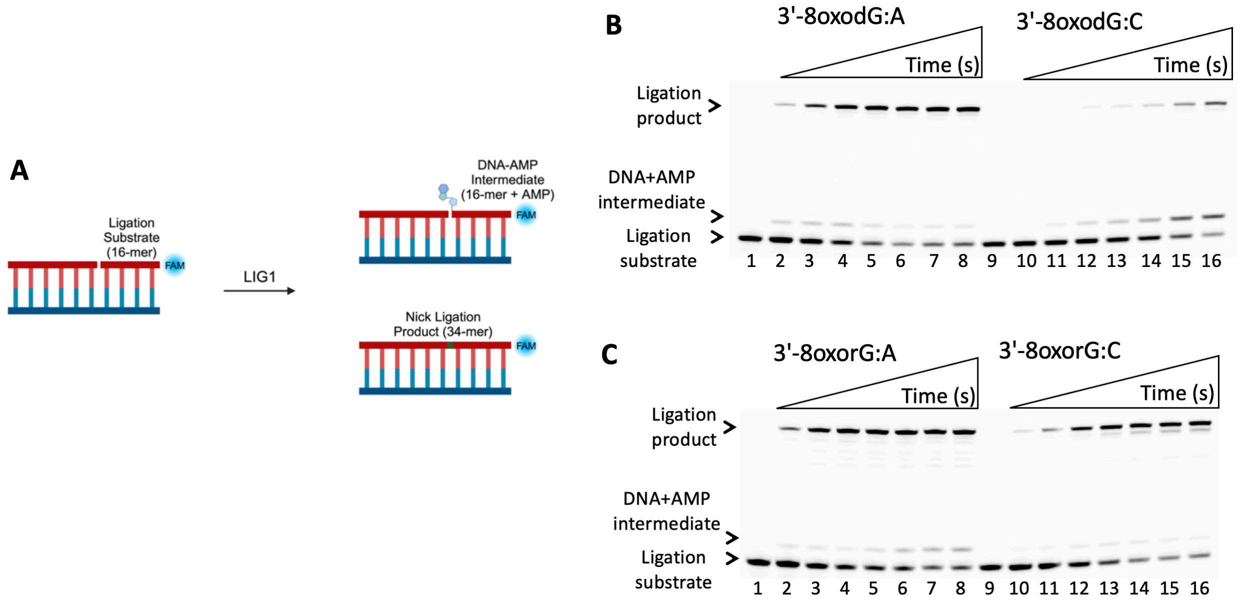

Supplementary Figure 12. Ligation of repair intermediates with 3'-preinserted 8oxoriboG. **(A)** Scheme showing the substrate and products observed in the ligation reaction. **(B)** Lanes 1 and 9 are the negative enzyme controls of the nick DNA substrates with template A or C, respectively. Lanes 2-8 and 10-16 are the ligation reaction in the presence of 3'-8oxodG:A and 3'-8oxodG:C and correspond to time points of 15, 30, 45, 60, 90, 180, and 300 sec. Representative gel image of three independent repeats. **(C)** Lanes 1 and 9 are the negative enzyme controls of the nick DNA substrates with template A or C, respectively. Lanes 2-8 and 10-16 are the ligation reaction in the presence of 3'-8oxorG:A and 3'-8oxorG:C and correspond to time points of 15, 30, 45, 60, 90, 180, and 300 sec. Representative gel image of three independent repeats. Graphs showing the time-dependent changes in the amount of ligation products of LIG1 are presented in Figure 4.

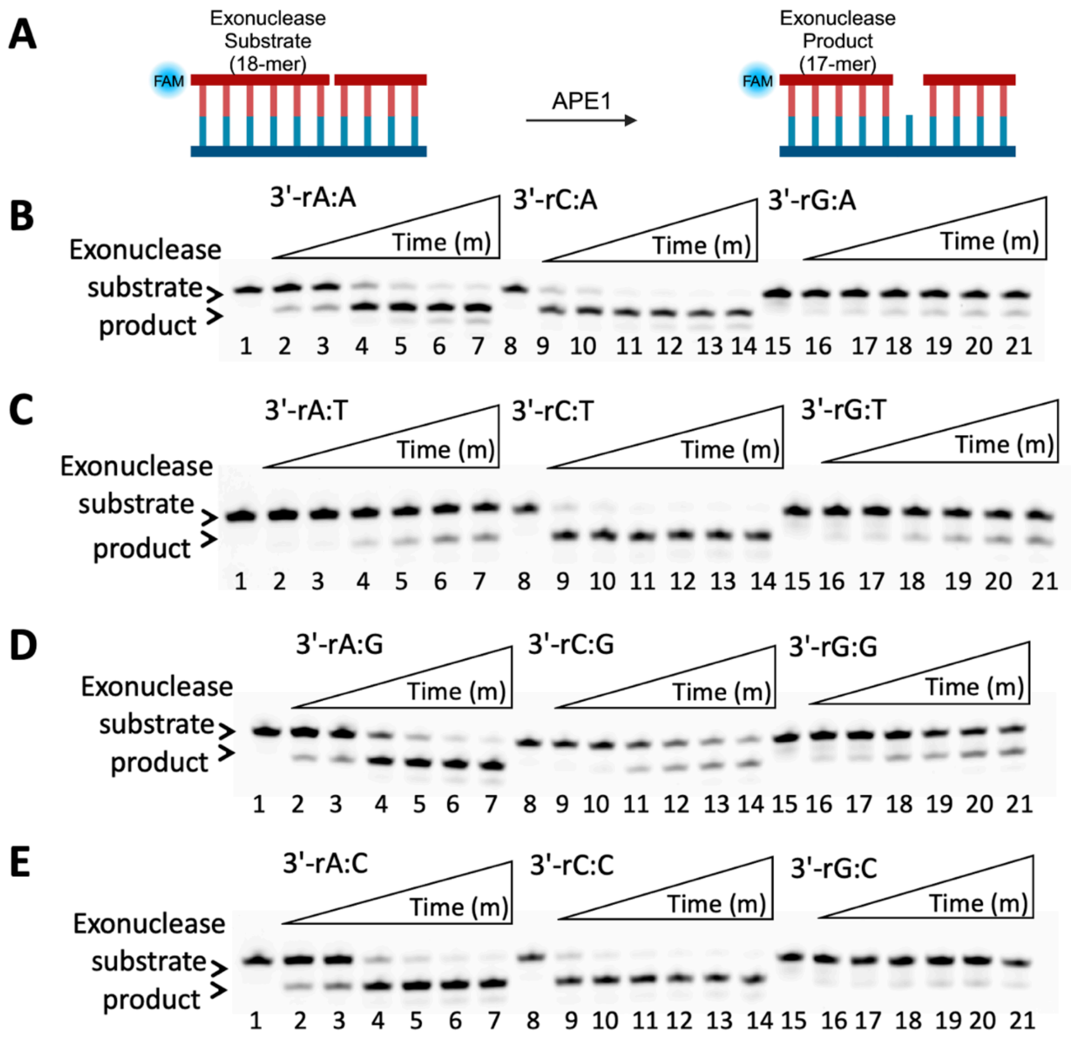

Supplementary Figure 13. Removal of 3'-ribonucleotide mismatches by APE1. (A) Scheme showing the substrate and product observed in the exonuclease assay. **(B-E)** Lanes 1, 8, and 15 are the negative enzyme controls of the nick DNA substrates with template A, T, G, and C. Lanes 2-7, 9-14, and 16-21 are the exonuclease removal products by APE1 in the presence of the 3'-ribonucleotide indicated in the figure, and correspond to time points of 0.5, 1, 3, 5, 8, and 10 min. Graphs showing the time-dependent changes in the amount of mismatch removal products of APE1 are presented in Figure 5.

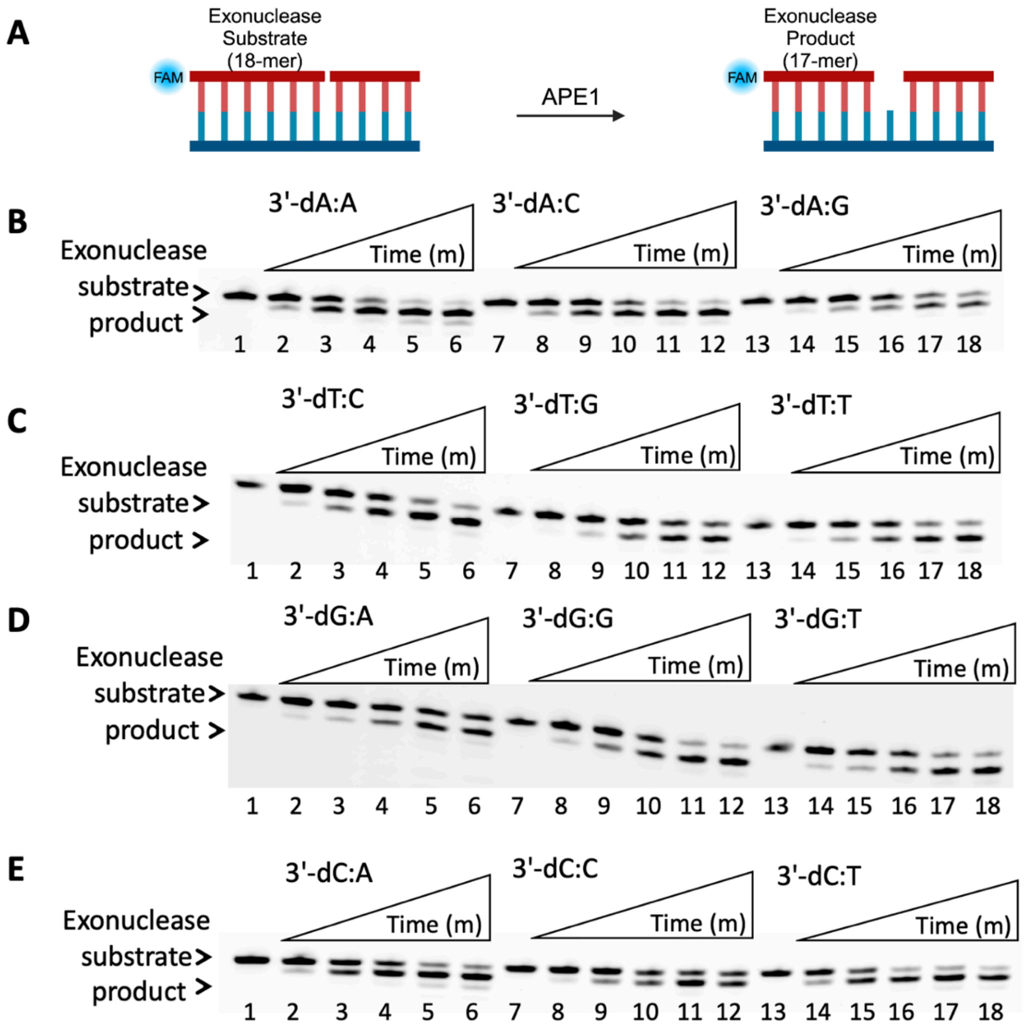

Supplementary Figure 14. Removal of 3'-mismatches by APE1. (A) Scheme showing the substrate and product observed in the exonuclease assay. **(B-E)** Lanes 1, 7, and 13 are the negative enzyme controls of the nick DNA substrates with 3'-dA, 3'-dT, 3'-dG, and 3'-dC. Lanes 2-6, 8-12, and 14-18 are the exonuclease removal products in the presence of 3'-mismatch by APE1, and correspond to time points of 0.5, 2, 4, 8, and 10 min. Graphs showing the time-dependent changes in the amount of mismatch removal products of APE1 are presented in Figure 6.

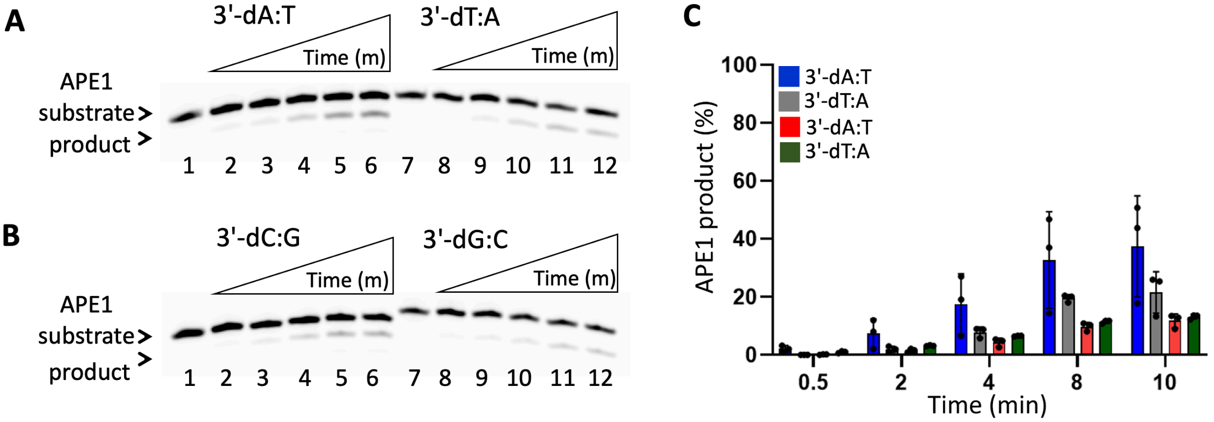

Supplementary Figure 15. Removal of 3'-bases from the nick DNA substrates containing Watson-Crick base paired ends by APE1. (A) Lanes 1 and 7 are the negative enzyme controls of the nick DNA substrates with template T and A, respectively. Lanes 2-6 and 8-12 are the exonuclease removal products in the presence of 3'-dA:T and 3'-dT:A by APE1, and correspond to time points of 0.5, 2, 4, 8, and 10 min. **(B)** Lanes 1 and 7 are the negative enzyme controls of the nick DNA substrates with template G and C, respectively. Lanes 2-6 and 8-12 are the exonuclease removal products in the presence of 3'-dC:G and 3'-dG:C by APE1, and correspond to time points of 0.5, 2, 4, 8, and 10 min. **(C)** Graph shows the time-dependent changes in the exonuclease removal products, the data are presented as the averages from three independent experiments ± SD.

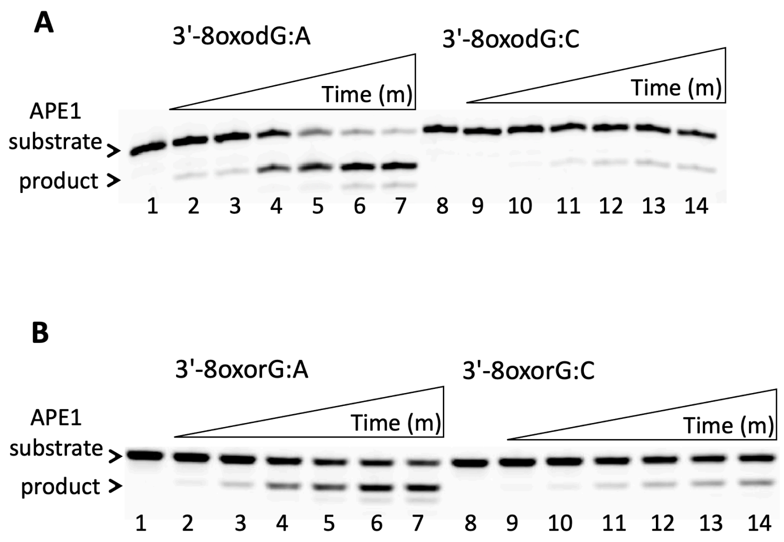

Supplementary Figure 16. Removal of 3'-8oxodG and 3'-8oxorG from the nick DNA substrates by APE1. (A-B) Lanes 1 and 8 are the negative enzyme controls of the nick DNA substrates with template A and C, respectively. Lanes 2-7 and 9-14 are the exonuclease removal products in the presence of 3'-8oxodG and 3'-8oxorG by APE1, and correspond to time points of 0.5, 1, 3, 5, 8, and 10 min. Graphs showing the time-dependent changes in the amount of mismatch removal products of APE1 are presented in Figures 5 and 6.

Gap DNA Substrates	Sequence
Template A	5'-FAM-CATGGGCGGCATGAACC GAGGCCCATCCTCACC-3'<br>3'-GTACCCGCCGTACTTGGACTCCGGGTAGGAGTGG-5'
Template C	5'-FAM-CATGGGCGGCATGAACC GAGGCCCATCCTCACC-3'<br>3'-GTACCCGCCGTACTTGGCCTCCGGGTAGGAGTGG-5'

Supplementary Table 1. Gap DNA substrates used in polβ nucleotide insertion assays. One nucleotide gap DNA substrates with template base A or C were used in nucleotide insertion assays. FAM denotes a fluorescent tag and is located at 5'-end of DNA substrates. The base at the template position is underlined.

Gap DNA Substrates	Sequence
Template A	5'-FAM-CATGGGCGGCATGAACC GAGGCCCATCCTCACC-FAM-3'<br>3'-GTACCCGCCGTACTTGGACTCCGGGTAGGAGTGG-5'
Template C	5'-FAM-CATGGGCGGCATGAACC GAGGCCCATCCTCACC-FAM-3'<br>3'-GTACCCGCCGTACTTGGCCTCCGGGTAGGAGTGG-5'

Supplementary Table 2. Gap DNA substrates used in coupled assays. One nucleotide gap DNA substrates with template base A or C were used in the coupled assays. FAM denotes a fluorescent tag and is located at both 3'- and 5'-ends of DNA substrates. The base at the template position is underlined.

Nick DNA Substrates	Sequence
3'-rA:A	5'-CATGGGCGGCATGAACC**A**GAGGCCCATCCTCACC-FAM-3'<br>3'-GTACCCGCCGTACTTGGACTCCGGGTAGGAGTGG-5'
3'-rG:A	5'-CATGGGCGGCATGAACC**G**GAGGCCCATCCTCACC-FAM-3'<br>3'-GTACCCGCCGTACTTGGACTCCGGGTAGGAGTGG-5'
3'-rC:A	5'-CATGGGCGGCATGAACC**C**GAGGCCCATCCTCACC-FAM-3'<br>3'-GTACCCGCCGTACTTGGACTCCGGGTAGGAGTGG-5'
3'-rA:T	5'-CATGGGCGGCATGAACC**A**GAGGCCCATCCTCACC-FAM-3'<br>3'-GTACCCGCCGTACTTGGTCTCCGGGTAGGAGTGG-5'
3'-rG:T	5'-CATGGGCGGCATGAACC**G**GAGGCCCATCCTCACC-FAM-3'<br>3'-GTACCCGCCGTACTTGGTCTCCGGGTAGGAGTGG-5'
3'-rC:T	5'-CATGGGCGGCATGAACC**C**GAGGCCCATCCTCACC-FAM-3'<br>3'-GTACCCGCCGTACTTGGTCTCCGGGTAGGAGTGG-5'
3'-rA:G	5'-CATGGGCGGCATGAACC**A**GAGGCCCATCCTCACC-FAM-3'<br>3'-GTACCCGCCGTACTTGGGCTCCGGGTAGGAGTGG-5'
3'-rG:G	5'-CATGGGCGGCATGAACC**G**GAGGCCCATCCTCACC-FAM-3'<br>3'-GTACCCGCCGTACTTGGGCTCCGGGTAGGAGTGG-5'
3'-rC:G	5'-CATGGGCGGCATGAACC**C**GAGGCCCATCCTCACC-FAM-3'<br>3'-GTACCCGCCGTACTTGGGCTCCGGGTAGGAGTGG-5'
3'-rA:C	5'-CATGGGCGGCATGAACC**A**GAGGCCCATCCTCACC-FAM-3'<br>3'-GTACCCGCCGTACTTGGCCTCCGGGTAGGAGTGG-5'
3'-rG:C	5'-CATGGGCGGCATGAACC**G**GAGGCCCATCCTCACC-FAM-3'<br>3'-GTACCCGCCGTACTTGGCCTCCGGGTAGGAGTGG-5'
3'-rC:C	5'-CATGGGCGGCATGAACC**C**GAGGCCCATCCTCACC-FAM-3'<br>3'-GTACCCGCCGTACTTGGCCTCCGGGTAGGAGTGG-5'
3'-8oxorG:A	5'-CATGGGCGGCATGAACC**X**GAGGCCCATCCTCACC-FAM-3'<br>3'-GTACCCGCCGTACTTGGACTCCGGGTAGGAGTGG-5'
3'-8oxorG:C	5'-CATGGGCGGCATGAACC**X**GAGGCCCATCCTCACC-FAM-3'<br>3'-GTACCCGCCGTACTTGGCCTCCGGGTAGGAGTGG-5'

Supplementary Table 3. Nick DNA substrates containing 3'-preinserted ribonucleotide mismatches used in DNA ligation assays. Nick DNA substrates with preinserted 3'-rA, rG, rC opposite template base A, T, G, or C were used in the ligation assays. FAM denotes a fluorescent tag and is located at 3'-end of DNA substrates. The base at the template position is underlined and the 3'-ribonucleotide is shown in bold. 8oxorG is represented by a X.

Nick DNA Substrates	Sequence
3'-dA:A	5'-CATGGGCGGCATGAACC**A**GAGGCCCATCCTCACC-FAM-3'<br>3'-GTACCCGCCGTACTTGGACTCCGGGTAGGAGTGG-5'
3'-dT:A	5'-CATGGGCGGCATGAACC**T**GAGGCCCATCCTCACC-FAM-3'<br>3'-GTACCCGCCGTACTTGGACTCCGGGTAGGAGTGG-5'
3'-dG:A	5'-CATGGGCGGCATGAACC**G**GAGGCCCATCCTCACC-FAM-3'<br>3'-GTACCCGCCGTACTTGGACTCCGGGTAGGAGTGG-5'
3'-dC:A	5'-CATGGGCGGCATGAACC**C**GAGGCCCATCCTCACC-FAM-3'<br>3'-GTACCCGCCGTACTTGGACTCCGGGTAGGAGTGG-5'
3'-dA:T	5'-CATGGGCGGCATGAACC**A**GAGGCCCATCCTCACC-FAM-3'<br>3'-GTACCCGCCGTACTTGGTCTCCGGGTAGGAGTGG-5'
3'-dT:T	5'-CATGGGCGGCATGAACC**T**GAGGCCCATCCTCACC-FAM-3'<br>3'-GTACCCGCCGTACTTGGTCTCCGGGTAGGAGTGG-5'
3'-dG:T	5'-CATGGGCGGCATGAACC**G**GAGGCCCATCCTCACC-FAM-3'<br>3'-GTACCCGCCGTACTTGGTCTCCGGGTAGGAGTGG-5'
3'-dC:T	5'-CATGGGCGGCATGAACC**C**GAGGCCCATCCTCACC-FAM-3'<br>3'-GTACCCGCCGTACTTGGTCTCCGGGTAGGAGTGG-5'
3'-dA:G	5'-CATGGGCGGCATGAACC**A**GAGGCCCATCCTCACC-FAM-3'<br>3'-GTACCCGCCGTACTTGGGCTCCGGGTAGGAGTGG-5'
3'-dT:G	5'-CATGGGCGGCATGAACC**T**GAGGCCCATCCTCACC-FAM-3'<br>3'-GTACCCGCCGTACTTGGGCTCCGGGTAGGAGTGG-5'
3'-dG:G	5'-CATGGGCGGCATGAACC**G**GAGGCCCATCCTCACC-FAM-3'<br>3'-GTACCCGCCGTACTTGGGCTCCGGGTAGGAGTGG-5'
3'-dC:G	5'-CATGGGCGGCATGAACC**C**GAGGCCCATCCTCACC-FAM-3'<br>3'-GTACCCGCCGTACTTGGGCTCCGGGTAGGAGTGG-5'
3'-dA:C	5'-CATGGGCGGCATGAACC**A**GAGGCCCATCCTCACC-FAM-3'<br>3'-GTACCCGCCGTACTTGGCCTCCGGGTAGGAGTGG-5'
3'-dT:C	5'-CATGGGCGGCATGAACC**T**GAGGCCCATCCTCACC-FAM-3'<br>3'-GTACCCGCCGTACTTGGCCTCCGGGTAGGAGTGG-5'
3'-dG:C	5'-CATGGGCGGCATGAACC**G**GAGGCCCATCCTCACC-FAM-3'<br>3'-GTACCCGCCGTACTTGGCCTCCGGGTAGGAGTGG-5'
3'-dC:C	5'-CATGGGCGGCATGAACC**C**GAGGCCCATCCTCACC-FAM-3'<br>3'-GTACCCGCCGTACTTGGCCTCCGGGTAGGAGTGG-5'
3'-8oxodG:A	5'-CATGGGCGGCATGAACC**X**GAGGCCCATCCTCACC-FAM-3'<br>3'-GTACCCGCCGTACTTGGACTCCGGGTAGGAGTGG-5'
3'-8oxodG:C	5'-CATGGGCGGCATGAACC**X**GAGGCCCATCCTCACC-FAM-3'<br>3'-GTACCCGCCGTACTTGGCCTCCGGGTAGGAGTGG-5'

Supplementary Table 4. Nick DNA substrates containing 3'-preinserted mismatches used in DNA ligation assays. Nick DNA substrates with preinserted 3'-dA, dT, dG, dC opposite template base A, T, G, or C were used in the ligation assays. FAM denotes a fluorescent tag and is located at 3'-end of DNA substrates. The base at the template position is underlined and the 3'-deoxyribonucleotide is shown in bold. 8oxodG is represented by a X.

Nick DNA Substrates	Sequence
3'-rA:A	5'-FAM-CATGGGCGGCATGAACC**A**GAGGCCCATCCTCACC-3'<br>3'-GTACCCGCCGTACTTGGACTCCGGGTAGGAGTGG-5'
3'-rG:A	5'-FAM-CATGGGCGGCATGAACC**G**GAGGCCCATCCTCACC-3'<br>3'-GTACCCGCCGTACTTGGACTCCGGGTAGGAGTGG-5'
3'-rC:A	5'-FAM-CATGGGCGGCATGAACC**C**GAGGCCCATCCTCACC-3'<br>3'-GTACCCGCCGTACTTGGACTCCGGGTAGGAGTGG-5'
3'-rA:T	5'-FAM-CATGGGCGGCATGAACC**A**GAGGCCCATCCTCACC-3'<br>3'-GTACCCGCCGTACTTGGTCTCCGGGTAGGAGTGG-5'
3'-rG:T	5'-FAM-CATGGGCGGCATGAACC**G**GAGGCCCATCCTCACC-3'<br>3'-GTACCCGCCGTACTTGGTCTCCGGGTAGGAGTGG-5'
3'-rC:T	5'-FAM-CATGGGCGGCATGAACC**C**GAGGCCCATCCTCACC-3'<br>3'-GTACCCGCCGTACTTGGTCTCCGGGTAGGAGTGG-5'
3'-rA:G	5'-FAM-CATGGGCGGCATGAACC**A**GAGGCCCATCCTCACC-3'<br>3'-GTACCCGCCGTACTTGGGCTCCGGGTAGGAGTGG-5'
3'-rG:G	5'-FAM-CATGGGCGGCATGAACC**G**GAGGCCCATCCTCACC-3'<br>3'-GTACCCGCCGTACTTGGGCTCCGGGTAGGAGTGG-5'
3'-rC:G	5'-FAM-CATGGGCGGCATGAACC**C**GAGGCCCATCCTCACC-3'<br>3'-GTACCCGCCGTACTTGGGCTCCGGGTAGGAGTGG-5'
3'-rA:C	5'-FAM-CATGGGCGGCATGAACC**A**GAGGCCCATCCTCACC-3'<br>3'-GTACCCGCCGTACTTGGCCTCCGGGTAGGAGTGG-5'
3'-rG:C	5'-FAM-CATGGGCGGCATGAACC**G**GAGGCCCATCCTCACC-3'<br>3'-GTACCCGCCGTACTTGGCCTCCGGGTAGGAGTGG-5'
3'-rC:C	5'-FAM-CATGGGCGGCATGAACC**C**GAGGCCCATCCTCACC-3'<br>3'-GTACCCGCCGTACTTGGCCTCCGGGTAGGAGTGG-5'
3'-8oxorG:A	5'-FAM-CATGGGCGGCATGAACC**X**GAGGCCCATCCTCACC-3'<br>3'-GTACCCGCCGTACTTGGACTCCGGGTAGGAGTGG-5'
3'-8oxorG:C	5'-FAM-CATGGGCGGCATGAACC**X**GAGGCCCATCCTCACC-3'<br>3'-GTACCCGCCGTACTTGGCCTCCGGGTAGGAGTGG-5'

Supplementary Table 5. Nick DNA substrates containing 3'-preinserted ribonucleotide mismatches used in APE1 exonuclease assays. Nick DNA substrates with preinserted 3'-rA, rG, rC opposite template base A, T, G, or C were used in the ligation assays. FAM denotes a fluorescent tag and is located at 5'-end of DNA substrates. The base at the template position is underlined and the 3'-ribonucleotide is shown in bold. 8oxorG is represented by a X.

Nick DNA Substrates	Sequence
3'-dA:A	5'-FAM-CATGGGCGGCATGAACC**A**GAGGCCCATCCTCACC-3'<br>3'-GTACCCGCCGTACTTGGACTCCGGGTAGGAGTGG-5'
3'-dT:A	5'-FAM-CATGGGCGGCATGAACC**T**GAGGCCCATCCTCACC-3'<br>3'-GTACCCGCCGTACTTGGACTCCGGGTAGGAGTGG-5'
3'-dG:A	5'-FAM-CATGGGCGGCATGAACC**G**GAGGCCCATCCTCACC-3'<br>3'-GTACCCGCCGTACTTGGACTCCGGGTAGGAGTGG-5'
3'-dC:A	5'-FAM-CATGGGCGGCATGAACC**C**GAGGCCCATCCTCACC-3'<br>3'-GTACCCGCCGTACTTGGACTCCGGGTAGGAGTGG-5'
3'-dA:T	5'-FAM-CATGGGCGGCATGAACC**A**GAGGCCCATCCTCACC-3'<br>3'-GTACCCGCCGTACTTGGTCTCCGGGTAGGAGTGG-5'
3'-dT:T	5'-FAM-CATGGGCGGCATGAACC**T**GAGGCCCATCCTCACC-3'<br>3'-GTACCCGCCGTACTTGGTCTCCGGGTAGGAGTGG-5'
3'-dG:T	5'-FAM-CATGGGCGGCATGAACC**G**GAGGCCCATCCTCACC-3'<br>3'-GTACCCGCCGTACTTGGTCTCCGGGTAGGAGTGG-5'
3'-dC:T	5'-FAM-CATGGGCGGCATGAACC**C**GAGGCCCATCCTCACC-3'<br>3'-GTACCCGCCGTACTTGGTCTCCGGGTAGGAGTGG-5'
3'-dA:G	5'-FAM-CATGGGCGGCATGAACC**A**GAGGCCCATCCTCACC-3'<br>3'-GTACCCGCCGTACTTGGGCTCCGGGTAGGAGTGG-5'
3'-dT:G	5'-FAM-CATGGGCGGCATGAACC**T**GAGGCCCATCCTCACC-3'<br>3'-GTACCCGCCGTACTTGGGCTCCGGGTAGGAGTGG-5'
3'-dG:G	5'-FAM-CATGGGCGGCATGAACC**G**GAGGCCCATCCTCACC-3'<br>3'-GTACCCGCCGTACTTGGGCTCCGGGTAGGAGTGG-5'
3'-dC:G	5'-FAM-CATGGGCGGCATGAACC**C**GAGGCCCATCCTCACC-3'<br>3'-GTACCCGCCGTACTTGGGCTCCGGGTAGGAGTGG-5'
3'-dA:C	5'-FAM-CATGGGCGGCATGAACC**A**GAGGCCCATCCTCACC-3'<br>3'-GTACCCGCCGTACTTGGCCTCCGGGTAGGAGTGG-5'
3'-dT:C	5'-FAM-CATGGGCGGCATGAACC**T**GAGGCCCATCCTCACC-3'<br>3'-GTACCCGCCGTACTTGGCCTCCGGGTAGGAGTGG-5'
3'-dG:C	5'-FAM-CATGGGCGGCATGAACC**G**GAGGCCCATCCTCACC-3'<br>3'-GTACCCGCCGTACTTGGCCTCCGGGTAGGAGTGG-5'
3'-dC:C	5'-FAM-CATGGGCGGCATGAACC**C**GAGGCCCATCCTCACC-3'<br>3'-GTACCCGCCGTACTTGGCCTCCGGGTAGGAGTGG-5'
3'-8oxodG:A	5'-FAM-CATGGGCGGCATGAACC**X**GAGGCCCATCCTCACC-3'<br>3'-GTACCCGCCGTACTTGGACTCCGGGTAGGAGTGG-5'
3'-8oxodG:C	5'-FAM-CATGGGCGGCATGAACC**X**GAGGCCCATCCTCACC-3'<br>3'-GTACCCGCCGTACTTGGCCTCCGGGTAGGAGTGG-5'

Supplementary Table 6. Nick DNA substrates containing 3'-preinserted mismatches used in APE1 exonuclease assays. Nick DNA substrates with preinserted 3'-dA, dT, dG, dC opposite template base A, T, G, or C were used in the ligation assays. FAM denotes a fluorescent tag and is located at 5'-end of DNA substrates. The base at the template position is underlined and the 3'-deoxyribonucleotide is shown in bold. 8oxodG is represented by a X.
